# Comparison of the Effects of UV-C Light in the Form of Flash or Continuous Exposure: A Transcriptomic Analysis on *Arabidopsis thaliana* L.

**DOI:** 10.3390/ijms252413718

**Published:** 2024-12-22

**Authors:** Seyed Mehdi Jazayeri, Jawad Aarrouf, Laurent Urban, Félicie Lopez-Lauri

**Affiliations:** Unité Propre de Recherche Innovante, ERIT Plant Science, Interactions and Innovation, Avignon Université, 301 Rue Baruch de Spinoza, 84140 Avignon, France; jawad.aarrouf@univ-avignon.fr (J.A.); laurent.urban@univ-avignon.fr (L.U.); felicie.lauri@univ-avignon.fr (F.L.-L.)

**Keywords:** cell cycle, heat shock proteins, maintenance protein complex, mitochondrial respiration, photosynthesis, plant immunity, priming, programmed cell death

## Abstract

Ultraviolet C (UV-C) flash treatment represents a promising method for priming plants. This study compared the effects of 1 s (flash) and 60 s (60 s) UV-C exposures on the transcriptome of *Arabidopsis thaliana* L. plants. A dose of 200 J m^−2^ delivered in one second was observed to effectively stimulate plant defenses without causing any adverse effects on plant health. A total of 3054 and 1865 differentially expressed genes (DEGs) were identified in the flash and 60 s treatments, respectively, in comparison to the control plants. Of these, 1131 were common to both treatments. The flash treatment affected a greater number of transcription factors (415 genes) than the 60 s treatment (254 genes), indicating more pronounced alterations in gene expression. The flash treatment resulted in a significant overexpression of heat shock proteins (HSPs), heat shock factors (HSFs), and their associated genes, which impacted oxidative stress, proteostasis, genome stability, cell survival, and thermotolerance. The majority of mitochondrial genes were found to be upregulated, while photosynthetic genes exhibited a downregulation. These expression patterns coordinate electron transport and crosstalk between the nucleus, chloroplasts, and mitochondria, eliciting an adaptive protective response to UV-C flash. Additionally, the flash treatment resulted in alterations to several genes involved in cell cycle regulation, division, and DNA replication. These included ATP BMMs, BRCA2 s, IQDs, kinesin complex, MCM complex, CYCs, and CDKs, which ultimately led to cell cycle arrest as a temporary preparation for subsequent conditions. The present study demonstrates that a 1 s exposure to UV-C induces distinctive plant responses through coordinated gene expression. The findings suggest that the flash treatment is an innovative method that triggers a unique cellular response, prioritizing repair mechanisms and potentially enhancing plant immunity, resilience, and priming. It can be used as a plant resistance inducer and stimulator.

## 1. Introduction

The detrimental effects of pesticides on the environment and human health are well-documented in the scientific literature [1]. In addition to the development of resistant or tolerant cultivars, the biological control of pests, and the creation of complex cropping systems that are inherent to being less vulnerable and more resilient in the face of aggressors and stress conditions, plant resistance inducers (PRIs) are becoming increasingly prevalent and of growing interest [2]. They represent an alternative not only to fungicides, but also to the controversial copper and sulfur treatments. In contrast to fungicides, PRIs typically function as a prophylactic rather than a therapeutic measure against pathogens. In addition to chemical and biological PRIs, physical PRIs (mainly light and mechanical stress) are attracting increasing interest due to their distinctive characteristics. These include the capacity to be deployed even in the presence of precipitation and wind, the absence of the need for formulation, and the absence of residues on plants or in the soil. Physical PRIs are not subject to the lengthy homologation procedures typically required for chemical agents, primarily due to their lack of toxicity to humans and the environment. It is important to note, however, that certain types of radiation, particularly UV radiation (UVR), require adherence to established safety protocols. Nevertheless, these regulations are relatively straightforward to define and adhere to [3].

The potential of some wavelengths to act as physical PRIs is now recognized [4], including blue and red light, as well as UV-B and UV-C radiation [5,6]. Ultraviolet C (UV-C) light with a wavelength of 280–200 nm was initially observed to stimulate plant defenses in tobacco against tobacco mosaic virus (TMV) [7]. Subsequently, UV-C light in the form of exposures lasting between less than one and several minutes has been demonstrated to be an effective means of stimulating plant defenses against fungal diseases in a range of crops [6]. However, the necessity for exposure periods of at least one minute renders the use of low-intensity UV-C light practical only under greenhouse conditions. As a physical PRI, the results of several studies have demonstrated that the application of UV-C light in a dose-responsive manner can induce resistance against microbial pathogens [8,9]. A study of the natural variation in Arabidopsis demonstrated that responses to UVR are associated with pathogen defense and pathogen-like responses [10]. As demonstrated in the case of *Xanthomonas campestris*, this approach can be employed for the control of pathogens [11]. Furthermore, Ledermann, et al. observed that UV-C light flashes can be employed to enhance plant defense against *Erysiphe necator* under vineyard conditions, thereby reducing the necessity for fungicide applications [12]. Other studies have demonstrated the efficacy of UVR in stimulating plant resistance and defense through signal transduction pathways that trigger salicylic acid (SA) and pathogen resistance (PR) genes [3,7,13]. Surprisingly, Aarrouf and Urban demonstrated that 1 s flashes of UV-C light, when repeated three times with several days between treatments, can stimulate plant defenses [14]. Furthermore, they observed that 1 s treatments are more effective than 1 min exposures for the same amount of cumulative energy (1 kJ m^−2^ per treatment) [14]. Indeed, Aarrouf et al. demonstrated that flashes of UV-C light are perceived by UVR8, the photoreceptor for UV-B light. Furthermore, their findings indicated that UVR8 can indeed perceive UV-C light at the used dose, provided that it is in the form of flashes (1 s) rather than continuous illumination (60 s) [15].

The pioneering observations of Aarrouf and Urban have established that flashes of UV-C light (less than 2 s) are capable of stimulating the defenses of lettuce and tomato against *Botrytis cinerea*, pepper against *Phytophthora capsici*, and grapevine against *Plasmopara viticola*. Furthermore, these flashes are more effective than conventional exposures (60 s) at the same energy density (J m^−2^) and wavelength. This suggests that UV-C light flashes could be employed as a physical PRI in field conditions [12,14]. It is noteworthy that high-intensity flashes of UV-C light do not necessitate frequent treatments when employed as a physical protectant agent. It has been demonstrated that daytime treatments using a tractor at normal speed, in conjunction with specific lamps, are sufficient for the purposes of this study [3,12]. Additionally, the necessity for a comprehensive plant treatment, as is the case with low-intensity UV-C, is negated by the presence of a robust systemic response elicited by high-intensity flashes. This concept, which has its roots in pharmacology, has been extended to human health, leading to the formulation of the maximal acceptable dose (MAD) for UV radiation [16]. Subsequently, further evidence has been amassed to corroborate the assertion that UV-C light flashes markedly enhance crop defenses in commercial production settings. Notable reductions in disease symptoms were observed in a variety of crops, including on strawberries, tomatoes, and roses, which had been inoculated with powdery mildew in greenhouse settings that closely resembled commercial greenhouses [3,14,17,18]. This phenomenon may be attributed to alterations in plant–microorganism interactions resulting from UV radiation [19]. Similarly, grapevines infected with either powdery or downy mildew in vineyards demonstrated significant reductions in disease symptoms following treatment with high-intensity UV-C light [12]. In all of the aforementioned trials, the full preventive effect was achieved through the administration of three treatments at seven- to fifteen-day intervals prior to inoculation or the spontaneous development of the disease. While the efficacy of UV-C light as a physical PRI has been demonstrated in a variety of settings, including field conditions, the specific characteristics of plant responses to flash versus continuous UV-C light exposure remain poorly understood [14]. Further research is required to gain a comprehensive understanding of the transcriptomic responses triggered by UV-C light flashes and to discern any differences that may exist compared to conventional one-minute treatments.

The objective of the present study was to compare the effects of UV-C light delivered in the form of exposures of either 1 s or 60 s for the same amount of energy delivered (dose) on the transcriptome of *A. thaliana*. The dose was selected based on the results of preliminary trials on *A. thaliana* which indicated that this was the maximal dose that could be administered without causing adverse effects to the plants. Following the administration of three one-second (flash) treatments and sixty-second treatments of UV-C light, transcriptomic analyses were conducted on the treated plants in comparison to the control plants.

## 2. Results

### 2.1. Differential Gene Expression Analysis

**Figure 1:**
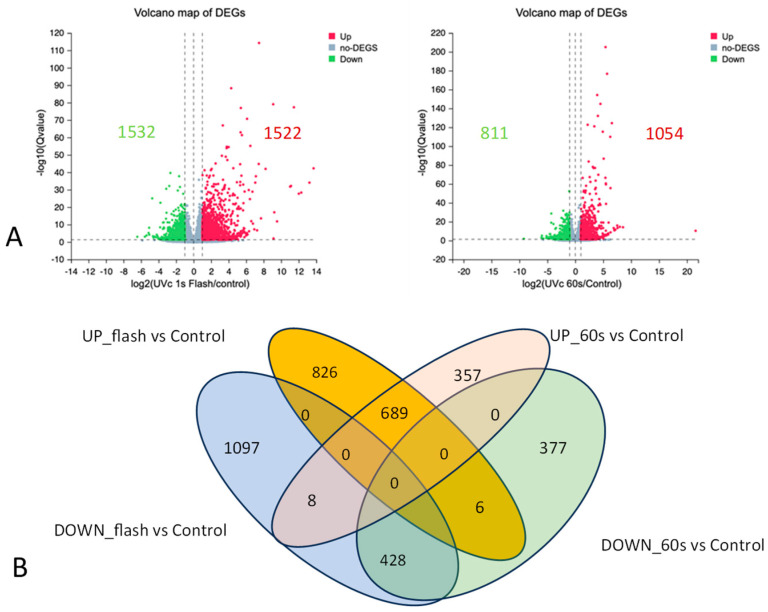
Analyses of differentially expressed genes (DEGs). (**A**) Volcano diagrams of DEGs identified in leaves after 4 h of UV-C flash (**left**) and UV-C 60 s treatments (**right**) vs. control. Spots above the threshold line (fold change cutoff ≥ 1 or fold change cutoff < −1, FDR < 0.05) indicate that differences are significant. Downregulated genes are displayed in green, while upregulated genes are displayed in red. Genes in grey are not DEGs. (**B**) Venn diagram representing the numbers of non-overlapped and overlapped DEGs in the four categories. ‘Up_1s flash vs. Control’ and ‘Down_1s flash vs. Control’ refer to upregulated and downregulated DEGs detected in 1 s UV-C (Flash) treated leaves compared to the control, respectively. ‘Up_60s vs. Control’ and ‘Down_60s vs. Control’ refer to upregulated and downregulated DEGs detected in 60 s UV-C treated leaves compared to control.

**Figure 2:**
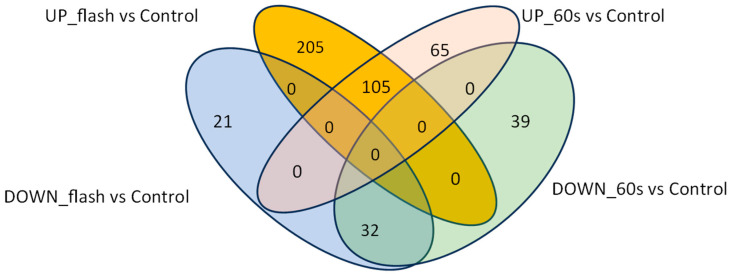
The comparisons of DEGs of the flash treatment and 60 s treatment based on log2FC of more than 3 or less than −3 and FDR ≤ 0.05. The number of specific upregulated DEGs with a fold change more than 10 for the flash treatment is 205, while there are 65 for the 60 s treatment. On the other hand, the specific downregulated DEGs are 21 and 39 for the flash and 60 s treatment, respectively. This suggests a higher gene activation rate by the flash treatment.

The RNA-seq data analysis compares the gene expression profiles of control plants with those of plants treated with either 1 s UV-C radiation (flash treatment) or 60 s UV-C radiation (60 s treatment). A total of 3054 differentially expressed genes (DEGs) are identified in the flash treatment group, while the 60 s treatment group exhibits 1865 DEGs (Figure 1). In particular, the flash treatment has 1532 overexpressed and 1522 underexpressed DEGs, while the 60 s treatment has 1054 overexpressed and 811 underexpressed DEGs. A total of 1031 DEGs are common to both treatments (Figure 1). Further analysis with a fold change greater than 10 or less than −10 (log2FC ≥ 3 or log2FC ≤ −3) reveals that 363 DEGs in the flash treatment exhibits an absolute fold change of 10, in comparison to 241 in the 60 s treatment (Figure 2).

To validate the RNA-seq data, five genes among the DEGs are tested with RT-qPCR. The selected genes include HSP18.1 (heat shock protein 18.1), HSFA2 (heat shock transcription factor A2), HSA32 (heat-stress-associated 32), and MEG1 and MEG2 (mitochondrial GrpE 1 and 2). Two housekeeping genes, ACT2 and UBQ9, are utilized, as their expression level remained unaltered by any of the treatments. The correlation between the RNA-seq and RT-qPCR data is strong, with a linear relationship evident for gene expression (R^2^ = 0.80) (Supplementary File). This serves to confirms that the data obtained from RNA-seq are in accordance with the results of RT-qPCR (Supplementary File).

### 2.2. Gene Ontology (GO) Enrichment Analysis

**Figure 3:**
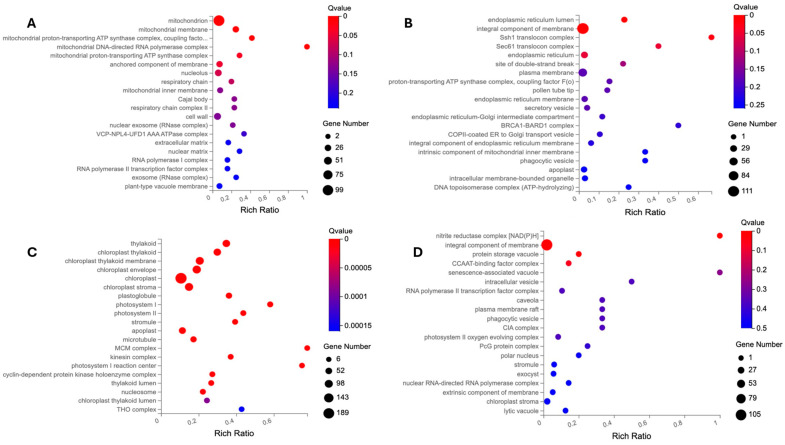
The cellular component (CC) of GO enrichment analysis. The up- and downregulated DEGs are employed as separate entities to undertake GO-based enrichment analysis. (**A**) The GO enrichment of upregulated DEGs of the flash treatment. (**B**) The GO enrichment of upregulated DEGs of the 60 s treatment. (**C**) The GO enrichment of downregulated DEGs of the flash treatment. (**D**) The GO enrichment of downregulated DEGs of the 60 s treatment. In the case of the flash treatment, the affected cellular components are related to the chloroplast, mitochondrion, cell wall, nucleus, extracellular matrix, and their related compartmentalization. The targeted cellular parts are the vesicle and vacuole, as well as endoplasmic reticulum. It is noteworthy that the GO terms related to the MCM complex, kinesin, and microtubule are specific to the flash treatment. This suggests that UV-C is able to penetrate cell structures effectively within a 1 s timeframe.

**Figure 4:**
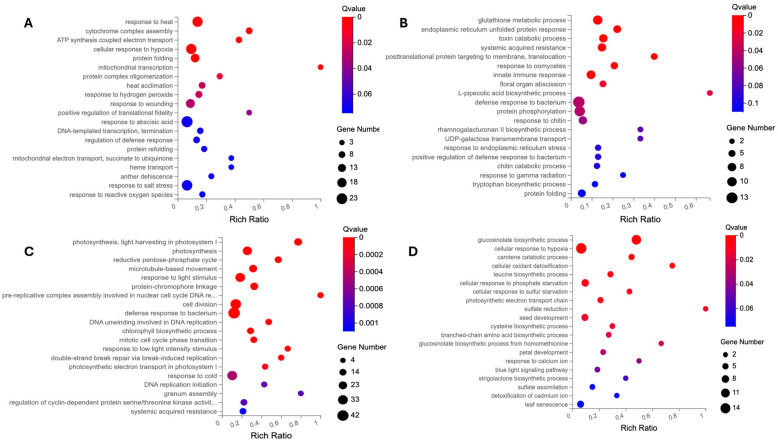
The biological process (BP) of GO enrichment analysis. The up- and downregulated DEGs were subjected to a GO-based enrichment analysis. (**A**) The GO enrichment of upregulated DEGs of the flash treatment. (**B**) The GO enrichment of upregulated DEGs of the 60 s treatment. (**C**). The GO enrichment of downregulated DEGs of the flash treatment. (**D**) The GO enrichment of downregulated DEGs of the 60 s treatment. The BP GO terms associated with the flash treatment pertain to thermotolerance, cell division, and transport. With regard to the 60 s treatment, the GO terms are associated with metabolite biosynthesis, responses to stresses, and detoxification.

**Figure 5:**
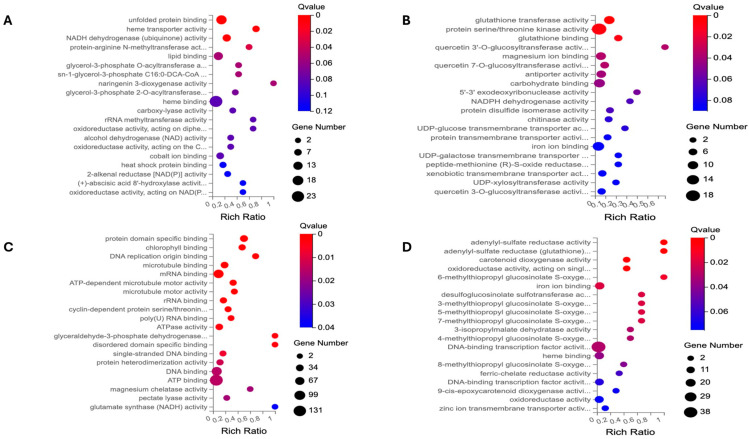
The molecular function (MF) of GO enrichment analysis. The up- and downregulated DEGs are employed as separate entities for the purpose of conducting a GO-based enrichment analysis. (**A**) The GO enrichment of upregulated DEGs of the flash treatment. (**B**) The GO enrichment of upregulated DEGs of the 60 s treatment. (**C**) The GO enrichment of downregulated DEGs of the flash treatment. (**D**) The GO enrichment of downregulated DEGs of the 60 s treatment. The GO terms of MF for the flash treatment are associated with DNA, RNA, protein, and chlorophyll. In the case of the 60 s treatments, the GO terms are assigned to enzymatic activities that are involved in metabolism of a variety of metabolites.

GO terms are employed to elucidate the biological mechanisms underlying DEGs and categorize them based on their compartmentalization, biological processes, and molecular functions. The GO enrichment of three GO categories, including cellular component (CC), biological process (BP), and molecular function (MF), is conducted separately for the two DEG lists of the flash and 60 s treatment (as illustrated in Figure 3, Figure 4 and Figure 5).

### 2.3. Functional Annotation of DEGs

#### 2.3.1. Metabolism and Regulation Genes

**Figure 6:**
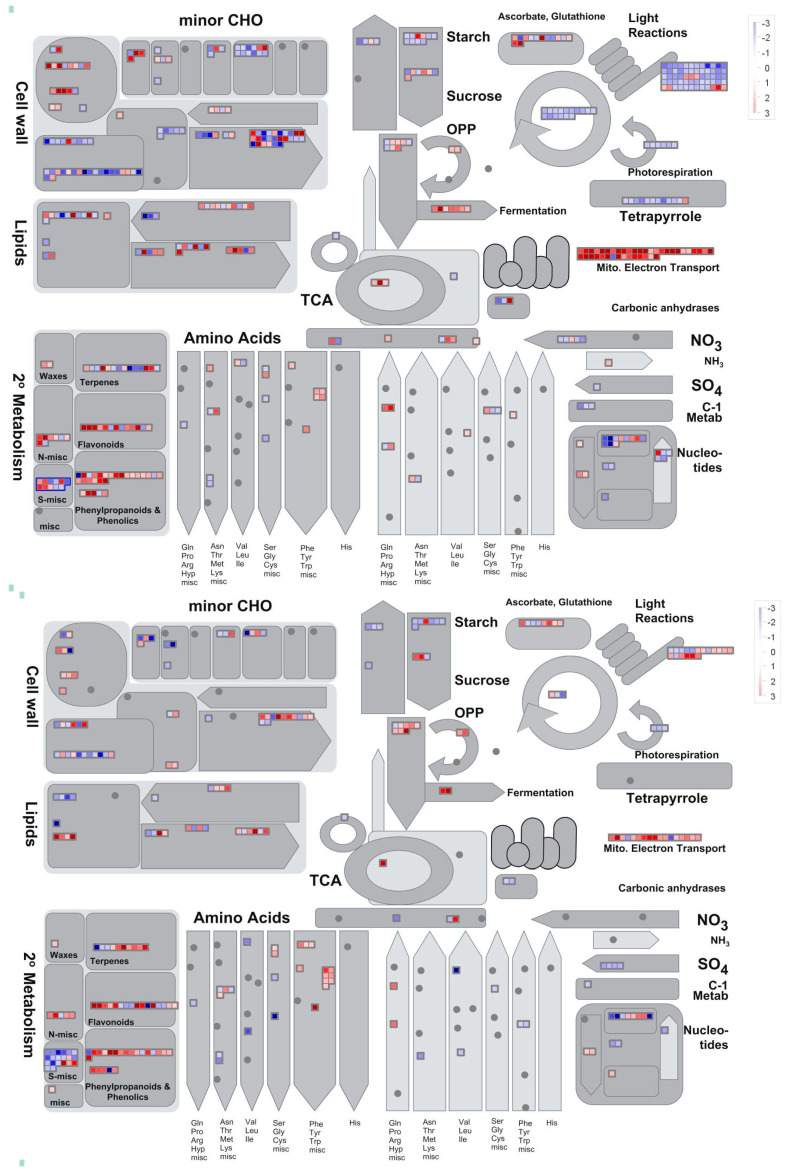
The metabolism overview pathway of MapMan. **Above**: the metabolism overview pathway of the flash treatment. **Below**: the metabolism overview pathway of the 60 s treatment. The differential effects of the flash and 60 s treatments on plants are evident from the higher number of differentially expressed genes (DEGs) observed in the former. The majority of sub-pathways within the metabolism overview demonstrate a greater number of DEGs in response to the flash treatment. However, the number of DEGs assigned to the amino acid metabolism section of Phe/Tyr/Trp/misc and the second metabolism of flavonoids and S-misc is greater for the 60 s treatment than for the flash treatment. Furthermore, the shared DEGs are illustrated in both maps for both treatments. The red dots indicate genes that are overexpressed, while the blue dots indicate genes that are underexpressed.

**Figure 7:**
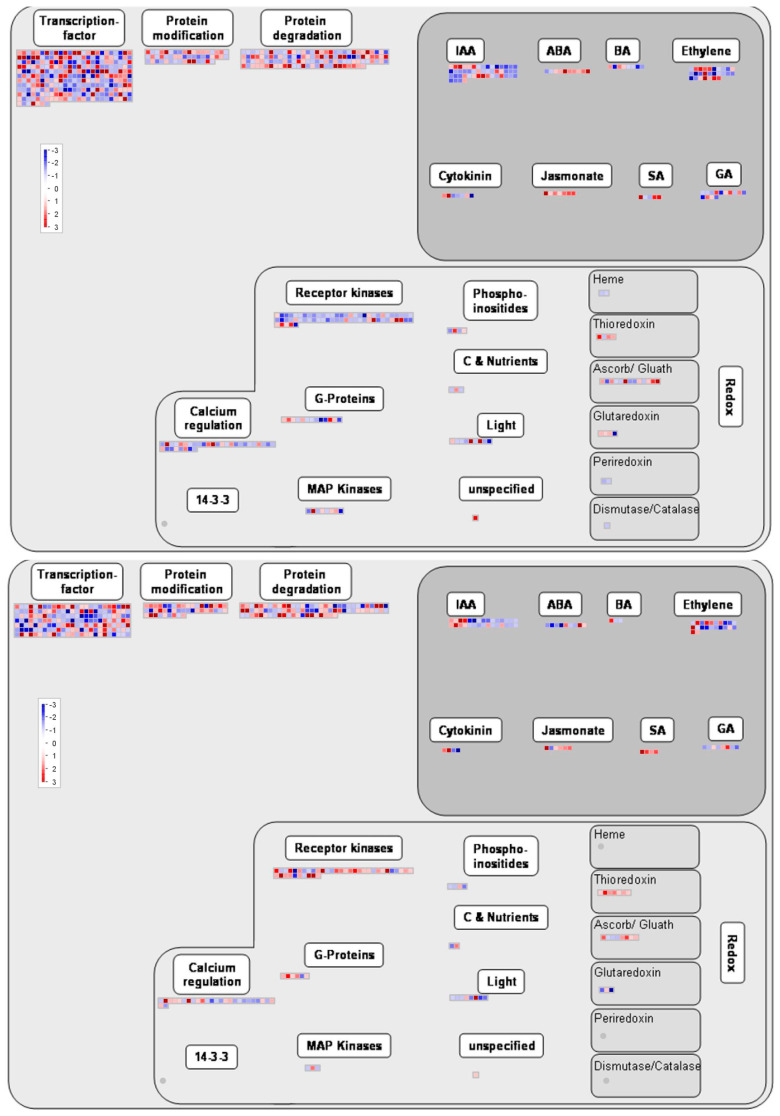
An overview of the regulatory pathway represented in MapMan. **Above**: the regulation overview pathway of the flash treatment. **Below**: the regulation overview pathway of the 60 s treatment. The differential effects of the flash and 60 s treatments on plants are evident from the higher number of DEGs observed in the former. All subcategories within the regulation overview exhibit a greater number of DEGs in response to the flash treatment. This discrepancy indicates a greater impact on the plant regulatory system affected with the flash treatment, suggesting that this treatment can stimulate downstream genes involved in transcription, regulation, and signaling, as well as protein modification and degradation. However, the flash treatment yielded a greater number of receptor kinases, with 65 DEGs, of which 51 were downregulated and 14 were upregulated. In contrast, the 60 s treatment resulted in the upregulation of 27 receptor kinase genes, while the 13 downregulated genes were associated with the 60 s treatment. Moreover, the genes that were differentially expressed in both treatments are illustrated in both maps. The red dots indicate genes that are overexpressed, while the blue dots indicate genes that are underexpressed.

**Figure 8:**
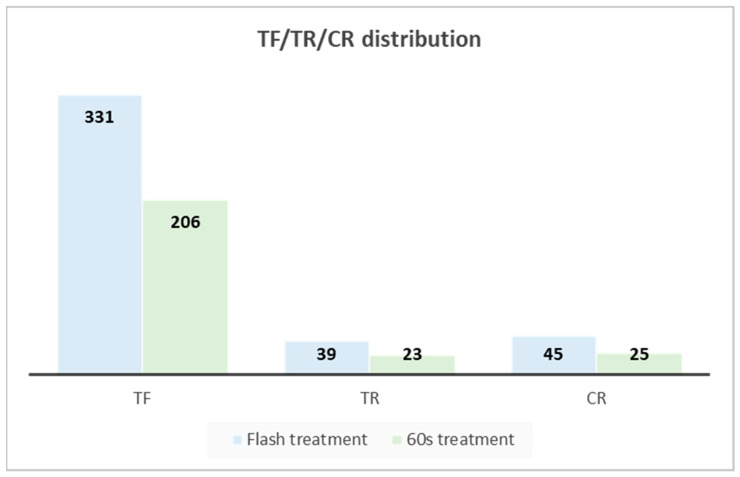
The transcription factors (TFs), transcription regulators (TRs), and chromatin regulators (CRs) for the flash (blue) and 60 s (green) treatments. The categorization was performed using the PlantTFcat tool. The number of regulatory and transcription system genes is 415 and 254, respectively, for the flash treatment and 60 s treatment. The greater number of DEGs within these three categories in the flash treatment group indicates that this treatment affects the transcriptional regulatory system in a more effective manner than the 60 s treatment. With respect to TFs and TRs, the flash treatment appears to exert a more pronounced influence on transcriptional regulation. With regard to CRs, the flash treatment prompts epigenetic occurrences and subsequently initiates priming.

To gain a deeper understanding of the general differences between the flash and 60 s treatments, the DEGs of both treatments are assigned to the metabolism (Figure 6) and regulation (Figure 7) overview pathways of MapMan. A total of 496 and 294 DEGs are assigned to the metabolism overview pathway for the flash and 60 s treatments, respectively. With the regards to the regulation overview pathway, 745 and 500 DEGs are assigned to this map for the flash and 60 s treatments, respectively. The difference of the number of DEGs assigned to these MapMan pathways is indicative of the fact that two treatments altered the expression of genes in different manners. As illustrated in Figure 8, the number of DEGs assigned to transcription factors (TFs), transcription regulators (TRs), and chromatin regulators (CRs) differs between the flash and 60 s treatments. The DEGs of the flash treatment are 412, distributed across three categories: TF, TR, and CR, within 58 families. Of these families, 18 are exclusive to the flash treatment. The number of DEGs assigned to these three categories is 254 for the 60 s treatment, distributed in forty-seven families, of which seven families are specific (Supplementary File). The results demonstrate that the flash treatment elicits a greater degree of gene expression than the 60 s treatment (Supplementary File).

#### 2.3.2. Photosynthetic and Mitochondrial Genes

**Figure 9:**
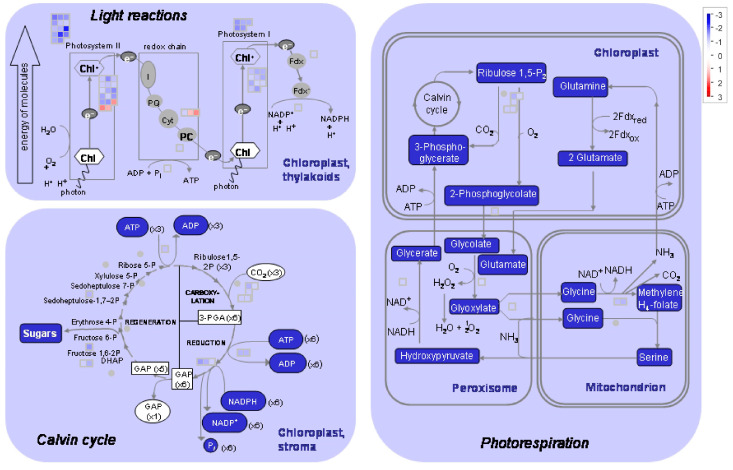

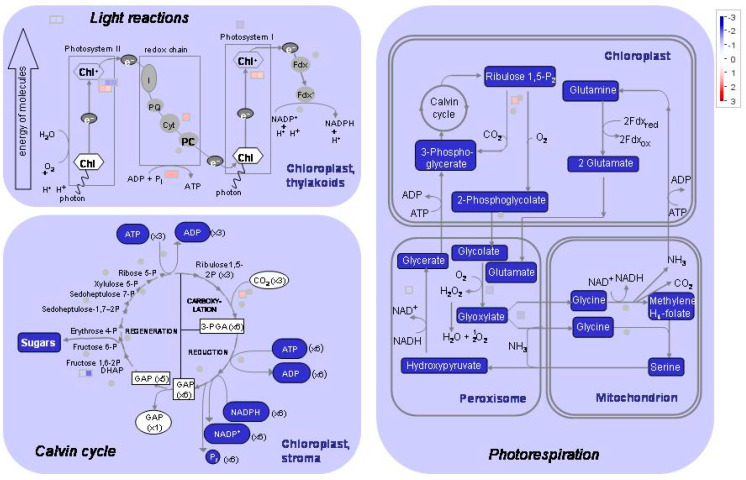
The MapMan photosynthesis pathway. The figure illustrates the differential expression of 82 and 20 genes in plants treated with the flash (**above**) and 60 s (**below**) treatments, respectively. The number of genes altered by the flash treatment is four times greater than that of the 60 s treatment. This indicates that the photosynthetic apparatus is more profoundly impacted by the flash treatment than by the 60 s treatment. In the case of the flash treatment, 31 and 16 DEGs are associated with PSII and PSI, respectively, while seven and three genes are associated with PSII and PSI, respectively. The genes of photorespiration comprise 12 and four in the flash and 60 s treatments, respectively. The genes that are overexpressed are indicated in red, while those that are underexpressed are indicated in blue.

**Figure 10:**
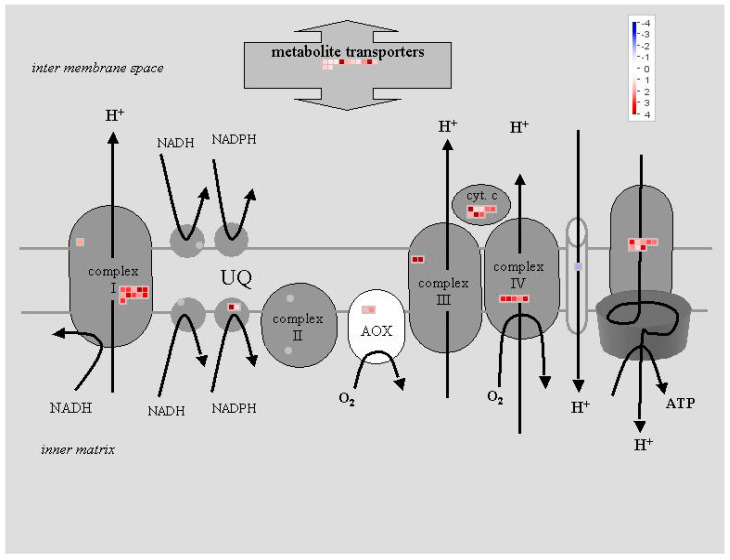

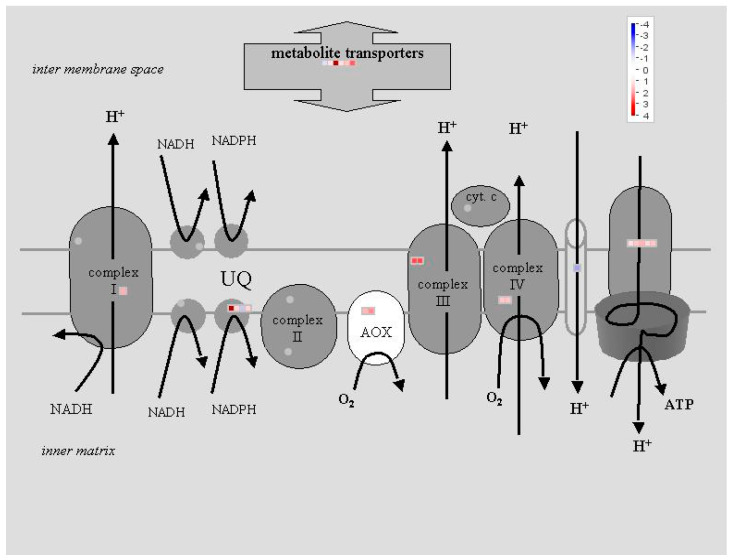
The MapMan mitochondrial electron transport pathway. The data indicate that there are 52 and 23 DEGs in plants treated with the flash (**above**) and 60 s (**below**) treatments, respectively. Similarly, the mitochondrial apparatus is more profoundly activated by the flash than by the 60 s treatment. Twelve genes associated with metabolite transporters exhibit differential overexpression in the flash treatment, with seven being exclusive to this treatment. For the 60 s treatment, six metabolite transporter genes are found. Eight genes associated with cytochrome c are overexpressed by the flash treatment, while no gene is altered by the 60 s treatment. For complex I of the electron transport, the flash treatment alters 11 genes, with 10 specific ones. The genes that are overexpressed are indicated in red, while those that are underexpressed are indicated in blue.

Table 1 lists the chloroplast genes, or ACTG genes, which include 21 genes. In the case of the flash treatment, twelve, three, and six ATCG genes are observed to undergo upregulation, downregulation, and no change, respectively. Of the twenty-one DEGs, fifteen are upregulated and six remain unaltered in the 60 s treatment. The DEGs associated with photosynthesis are assigned to 82 and 20 genes of the MapMan photosynthesis pathway for the flash and 60 s treatments, respectively (Figure 9 and Supplementary File). The mitochondrial DEGs are revealed in Table 2, comprising 79 DEGs associated with mitochondrial activity, and Table 3, encompassing 45 DEGs of the mitochondrial genome (referred to as ATMG genes). In the flash treatment, 71 and 8 genes identified as being related to mitochondrial activity are thus classified as upregulated and downregulated genes, respectively. Among the seventy-nine mitochondrial genes, the 60 s treatment alters the expression of three genes, while the remaining genes remain unaltered. Of the 45 identified ATMGs, all were found to be upregulated by the flash treatment, while 14 of these were also upregulated by the 60 s treatment. The absence of downregulated genes among the ATMG genes suggests a direct stimulating effect of UV-C light on mitochondrial activities. The mitochondrial electron transport pathway of MapMan (Figure 10) demonstrates that 52 and 23 DEGs are involved in the electron transport pathway for the flash and 60 s treatments, respectively. Of these 52 DEGs, 32 were exclusive to the flash treatment. Only three DEGs were specific to the 60 s exposure, while the remaining twenty DEGs were shared with the flash treatment.

#### 2.3.3. Programmed Cell Death (PCD) Genes

The DEGs associated with PCD are presented in Table 4. A total of 32 DEGs associated with PCD are identified in both treatments. Among these, 29 genes exhibited altered expression in response to the flash treatment, while three DEGs were specifically affected by the 60 s treatment but not by the flash treatment. In total, the 60 s treatment alters the expression of 16 genes and leaves 16 genes unchanged. These genes play roles in various processes that regulate PCD and its associated mechanisms such as cell survival, apoptosis, signaling, transcription regulation, stress responses, and development.

#### 2.3.4. Heat Shock Protein (HSP) and Heat Shock Factor (HSF) Genes

The list of HSPs, HSFs, and their related genes are found in Table 5. A total of 56 genes are retrieved for both treatments. Among these, seven DEGs exhibit decreased gene expression levels in the flash treatment, while forty-five demonstrate increased expression and four remained unaltered. In the case of the 60 s treatment, 29 DEGs are identified, while the expression of 27 genes remains unchanged (i.e., they are specific to the flash treatment). The 60 s treatment, respectively, upregulated and downregulated twenty-five and four genes. The specific DEGs of the 60 s treatment are four, comprising two upregulated and two downregulated genes (Supplementary File). With regard to HSPs, 50 and 29 DEGs related to heat shock proteins (HSPs) or HSP-like proteins were identified in response to the flash and 60 s treatments, respectively (Table 5).

#### 2.3.5. Cell Cycle and DNA Replication and Repair Genes

Table 6 enumerates the genes implicated in the processes of the cell cycle and DNA replication and repair. In total, 71 DEGs are classified as pertaining to the cell cycle and DNA replication. Of seventy-one DEGs, eight exhibit an upregulated expression level, while fifty-nine demonstrate downregulated expression in response to the flash treatment. The differential expression of genes in response to the 60 s treatment is observed in five upregulated and three downregulated genes, respectively. Conversely, four and sixty-three genes are unaltered by the flash and 60 s treatments, respectively. In total, 24 genes encoding kinesin proteins are altered by both treatments (Supplementary File). Among these, one is found to be upregulated and twenty-two are found to be downregulated by the flash treatment. One kinesin-like gene (AT3G13229) is observed to be downregulated in the 60 s treatment while remaining unchanged in the flash treatment. The rest of the kinesin proteins are unchanged in the 60 s treatment. In total, the expression of nine IQD genes is altered by the flash treatment, with one exhibiting upregulated expression and eight demonstrating a downregulated expression level. Of these nine genes, the expression of two IQD genes is altered by the 60 s treatment (one upregulated and one downregulated) and the remaining seven remain unchanged (Supplementary File).

#### 2.3.6. Cell Wall Genes

A total of 198 DEGs are assigned to the cell wall using MapMan. For the flash treatment, 93 genes are underexpressed, 76 are overexpressed, and 29 remain unchanged. Interestingly, the genes that remain unaltered by the flash treatment exhibit modifications following the 60 s treatment. In contrast, the 60 s treatment results in 36 genes being underexpressed, 45 being overexpressed, and 117 remaining unchanged (Supplementary File). The data suggest that the flash treatment exerts a more pronounced effect on cell wall integration than the 60 s treatment, potentially enhancing the penetration of UV-C radiation in a 1 s flash.

## 3. Discussion

### 3.1. Distinct Gene Expression Responses to Treatments by Either 1 s UV-C Flashes vs. 60 s Continuous Exposures

The results demonstrated a notable divergence in gene expression between the two UV-C treatments, with 1923 and 734 DEGs specific to the flash and 60 s treatments, respectively. Furthermore, 1131 DEGs were common to all treatments (Figure 1), indicating general responses to UV-C radiation irrespective of exposure duration. Plants are not typically exposed to UV-C radiation and have evolved mechanisms to mitigate its effects. The common DEGs participate in a variety of biological processes, including stress response, transcription factors, photosystem function, mitochondrial electron transport, amino acid metabolism, secondary metabolism, hormone metabolism, protein modification, signaling, transporter activity, RNA regulation, and DNA metabolism. These processes collectively orchestrate responses to UV radiation [20].

In case of UV-C perception by both treatments, UVR8 does not appear to play a role in the perception of UV-C, regardless of whether it is delivered in 60 s or in the form of flash. This is based on the observation that none of the known molecular markers linked to UVR8 signaling were identified among DEGs in this trial. The UV-C flashes are at the origin of an original signaling system that remains to be elucidated. Moreover, it is conceivable that the UV-C dose utilized in this study may be absorbed by additional, as yet unidentified UV receptors. In this case, several DEGs with high expression levels whose function is not known were identified, which may serve as potential candidates for UV-C perception (Supplementary File). Furthermore, among the commonly differentially expressed genes between the two treatments, several receptor genes with overexpression were identified, including LRR XI-23 (leucine-rich receptor-like protein kinase), ERD2 (endoplasmic reticulum retention defective 2), SD1-29 (S-domain-1 29), FRK1 (FLG22-induced receptor-like kinase 1), RLP24 (receptor-like protein 24), RLP30, AT3G22060, CRK13 (cysteine-rich RLK (RECEPTOR-like protein kinase) 13), and LECRKA4 (lectin receptor kinase a4.1). Additionally, AT5G11410 and AT5G60650 were identified. These genes are associated with defense and immunity as PAMPs, which leads us to propose that they serve as receptors upon UV-C radiation. However, exploring the possible involvement of proteins, such as those carbonylated under the oxidative stress associated with UV-C absorption in the form of flashes, could disclose whether they are receptors of UV-C.

Conversely, a number of particular genes were identified for each treatment with a high expression level, the function of which is currently unknown. It is therefore proposed that these genes may serve as candidate markers for the revelation of the underlying mechanistic differences between the two treatments and for the investigation of how treated plants may perceive and initiate downstream responses to UV-C light based on its intensity and duration in the flash form. A number of genes exhibited no expression in the 60 s treatment, whereas high expression was observed in the flash treatment. The genes in question are AT1G12040 (LRX1), AT5G09370 (bifunctional inhibitor/lipid-transfer protein/seed storage 2S albumin superfamily protein), AT5G13205, CUL2 (AT1G02980), and DAF1 (AT3G62230). Furthermore, transposable element genes, including AT1G21945, AT5G33303, AT1G41835, AT4G34300, and AT2G04135 were identified among the specific DEGs of the flash treatment. Of these, LRX1 and CUL2 are known receptors. LRX1 encodes a chimeric leucine-rich repeat/extensin protein that plays a regulatory role in root hair morphogenesis and elongation. CUL2 is a member of the Cullin family and functions as a scaffold protein for Elongin B and C, Rbx1, and various substrate recognition receptors, thereby forming E3 ubiquitin ligases. The functionality of the remaining DEGs is not yet fully understood and requires further investigation. However, we hypothesize that they may play a role in perception and initiating responses in the treated plants by UV-C flashes. This hypothesis requires further testing and validation through additional research.

The discrepancy between the treatment-specific DEGs (Figure 1 and Figure 2, Supplementary File) and the GO analysis for the CC, BP, and MF categories (Figure 3, Figure 4 and Figure 5) indicates that the flash treatment specifically impacted the expression of genes involved in cellular compartmentalization, particularly the cell wall, membrane, chloroplast, mitochondrion, and nucleolus, in comparison to the 60 s treatment. For example, 389 and 441 DEGs of the flash treatment were assigned to the GO terms “chloroplast” and “plastid”, respectively, whereas no such genes were identified in the 60 s treatment. The “mitochondria” GO term exhibited 275 DEGs for the flash treatment and no observed DEGs for the 60 s treatment. The cell wall was identified as a GO term for 95 and 55 DEGs of the flash and 60 s treatments, respectively. In contrast, the 60 s treatment exhibited a GO term “endoplasmic reticulum lumen” with 17 DEGs, while the flash treatment demonstrated no such occurrences. This indicates that the same UV-C intensity (200 J m^−2^) penetrates rapidly and effectively in one second, in contrast to the 60 s required for the other treatment [14].

In consideration of the diverse gene families and mechanisms, including those related to the cell cycle, DNA repair, defense responses, transporters, receptors, resistance genes, signaling, phytohormones, transcription factors, energy-related elements, and epigenomic components, the flash treatment is demonstrably more effective than the 60 s treatment in driving changes. The higher number and marked difference in DEGs indicate that the brief, pulsed 1 s UV-C radiation elicits a substantially different and potentially more impactful transcriptional response in plants compared to prolonged 60 s UV-C exposure. Therefore, a 1 s pulse of UV-C, at the same dosage, may prove to be a more efficacious approach than a longer exposure period in terms of perception, penetration, and subsequent responses.

As demonstrated by GO enrichment analysis, there is a discernible difference in UV-C perception between plants subjected to the two treatments. However, the proteins associated with UV-C light perception remain unclear. It is noteworthy that the genes with a high expression level that may serve as potential receptors have yet to be identified, as the functionality of almost all of them is not yet known. Nevertheless, we postulate that the highly expressed genes with unknown functions whose expression levels differ between the two treatments may serve as UV-C receptors, whether the plants are exposed to a brief, sudden pulse (1 s) or to a prolonged exposure (60 s). This hypothesis requires further investigation through functional genomics analysis of the candidate genes. Furthermore, the flash treatment demonstrated heightened activity in functional, structural, and regulatory aspects, indicating a more potent bioactive effect. This is concluded from 205 vs. 65 DEGs with a fold change greater than 10 for the flash and 60 s treatments, respectively (Figure 2 and Supplementary File). However, this remains to be elucidated in further research. The most highly expressed genes in the flash treatment include several heat shock proteins (HSPs) (Table 5), EGY3, SKS13, APRR6, and FTSH6, as well as several unidentified genes (Supplementary File). Many of these genes are located in the extracellular region and membrane, indicating that they are involved in the activation processes that facilitate the perception and penetration of UV-C light. These genes are involved in a number of processes (Figure 6 and Figure 7), including protein modification, cell wall formation, ROS homeostasis, signaling, and the stimulation of defense and growth mechanisms [21,22,23,24,25]. The elevated expression levels of these genes provide evidence to the hypothesis that the flash treatment stimulates plants through mechanisms that enhance defense by genes with multifunctionalities in growth and signaling. The genes that were highly and lowly expressed in the 60 s treatment indicate that prolonged UV-C exposure acts as a stressor. In contrast, the flash treatment appears to induce protective responses in a hormetic manner [14].

### 3.2. Transcriptional Regulation and Epigenetic Modifications Elicited by the Flash Treatment

The flash treatment demonstrated a more pronounced effect on the transcriptional system than the 60 s treatment (Figure 8 and Supplementary File), stimulating transcriptional and chromatin regulation, epigenetic remodeling, and signal transduction with greater efficacy. The greater number and functionality of DEGs in the flash treatment, including but not limited to C2H2, AP2-EREBP, MYB, and WRKY, suggest that a 1 s UV-C flash enhances perception, signaling, and penetration effects. This treatment also induced epigenetic regulation, which may contribute to plant priming and resilience.

The higher expression of TF/TR/CR families, like C2H2, AP2-EREBP, MYB, and WRKY (Supplementary File), involved in hormone signaling suggests that 1 s UV-C light enhanced perception, signaling, and penetration effects and highlights mechanisms related to PCD, signaling, ROS production and regulation, the cell cycle, and DNA repair and replication. They are among the most highly represented transcription factors (TFs) in plants and are involved in a number of biological mechanisms, such as regulation, signaling, stress responses, and adaptation [26,27]. Furthermore, the flash treatment resulted in the altered expression of genes, such as various histone H genes, HTR12, histone deacetylases (HDAs), and histone H3K4-specific methyltransferase (Supplementary File), which are involved in epigenetic mechanisms including DNA methylation, histone modifications, and histone variant deposition [28]. CRs interact with other epigenetic mechanisms, including DNA methylation, histone modifications, and histone variant deposition [29]. Notably, the flash treatment induced epigenetic regulation, potentially involved in plant priming and resilience.

All TIFY and JAZ (jasmonate-zim-domain protein) TFs, including AT1G30135 (JAZ8. TIFY5A), AT1G70700 (JAZ9. TIFY7), AT3G17860 (JAZ3. TIFY6B), and AT5G13220 (JAZ10), were found to be overexpressed as a result of the flash treatment (Supplementary File). These TFs function as transcriptional repressors within the JA signaling pathway. Additionally, the coronatine induced 1 (CORI3) gene, implicated in the JA signaling pathway [30], was also overexpressed by the flash treatment. It is known to contribute to plant defense mechanisms [31,32]. Furthermore, the flash treatment resulted in the overexpression of JA and JAZ TFs, along with genes such as APX and AOC. These TFs play pivotal roles in regulating growth and defense signaling pathways [33]. This suggests that the flash treatment stimulated a response related to jasmonates.

High mobility group (HMG) TFs (Supplementary File) are involved in DNA transcription, repair, and condensation. They regulate cell cycle genes such as CDKs through various mechanisms, including direct DNA binding and chromatin remodeling or the neutralization of other transcription factors involved in cancer signaling pathways. Their expression is correlated with cell cycle arrest [34]. As damage-associated molecular pattern (DAMP) proteins, they interact with extracellular receptors in response to inflammatory diseases and the immune system [35]. The expression of HMGs and related genes such as cell cycle CDKs was found to be downregulated by the flash treatment, which may indicate a potential role in cell cycle arrest. Further functional genomics studies are required to elucidate the mechanisms by which at a hormetic dose of UV-C light flash [14] stimulates priming and epigenetic modifications in plants.

### 3.3. The UV-C Flash Treatment Resulted in Remodeling of Energy Fluxes

The results demonstrate that at 200 J m^−2^, the downregulation of photosynthetic genes is specific to the 1 s UV-C flash. The 1 s UV-C flash had a more pronounced influence on photosynthesis compared to the 60 s treatment (Figure 9 and Supplementary File), downregulating key genes such as chlorophyll a-b binding, photosystem I and II reaction centers, light-harvesting complex, and Rubisco small subunits (Supplementary File). Additionally, the gene expression of magnesium chelatase (CHLD), chlorophyll synthase (CHLG), and phosphoglycerate kinase (PGK) was found to be downregulated. This indicates that the photosynthetic apparatus responds differently to 1 s and 60 s UV-C exposures. Notably, flashes of UV-C light have been observed to decrease gene expression associated with energy capture [36], thereby reducing the risk of photooxidative stress and photodamage from an imbalance between energy capture and use [37].

In contrast, genes encoding chloroplast proteins, including MATK, PSBA, PSBD, PSBC, ACCD, PETB, PETD, NDHD, NDHG, and NDHA, exhibited increased expression in response to both UV-C treatments (Table 1 and Supplementary File). This upregulation, in conjunction with the downregulation of other photosynthetic genes, serves to enhance the protective effects of the flash treatment against photooxidative stress. NDH genes encode the complex I (Figure 10), which is vital for electron transport during photosynthesis. They also influence signaling and defense pathways as redox sensors [38]. The NDH complex plays a role in maintaining redox balance, which is essential for plant immunity. It also balances ROS. This ROS-based response activates defense genes against pathogens and stress [39] and they influence signaling and defense pathways and balances essential for plant immunity [40]. It can be postulated that the redox status of plastoquinones may indicate the disruption of PSA and PSB by UV-C radiation [41]. PETB and PETD, which are components of PSII, facilitate the transfer of electrons from water to plastoquinone. The upregulation of NDH and PET genes has been observed to increase plastoquinone levels, thereby enhancing electron exchange between chloroplasts and mitochondria [42,43]. This process serves to balance electron flow and cellular energy, protect against ROS, and act as a signaling mechanism [44]. The flash treatment modifies the exchange of energy and electron between chloroplasts and mitochondria by changing the expression of genes associated with electron flow, ROS, and retrograde signaling.

The flash treatment resulted in a notable overexpression of ATMG genes, with the majority of mitochondrial DEGs exhibiting overexpression (with the exception of eight out of one-hundred and sixteen) (Table 2 and Table 3 and Figure 10). Five genes were found to be commonly expressed between the flash and 60 s treatments, with no treatment-specific ATMG genes identified for the 60 s treatment. Two of these genes are NAD genes, and another is uncoupling mitochondrial protein 5 (UCP5), which encodes dicarboxylate carriers involved in mitochondrial transport. In the TCA cycle, 13 NAD(H) dehydrogenases from the NAD gene family demonstrated elevated expression levels following the UV-C flash treatment, while four NDB family members and one NAD gene exhibited overexpression in the 60 s treatment (Figure 10, Supplementary File). This common overexpression indicates that UV-C affects plant mitochondrial genes independently of exposure duration. This is likely due to the fact that UV-C serves as a resource of light energy, stimulating mitochondria as organelles that regulate cellular energy and the production of reactive oxygen species (ROS), free radicals, and electron transport [45]. However, the flash treatment yielded a distinct pattern of mitochondrial gene overexpression, suggesting active remodeling of energy metabolism by short UV-C radiation. This remodeling via the electron transport chain and higher mitochondrial activity optimizes energy production, regulates ROS levels, and maintains cellular redox balance and homeostasis, ultimately enhancing plant health [46,47].

Genes involved in energy-related metabolism, such as glycolysis, the TCA cycle, and ATP biosynthesis, are upregulated by biotic stress and UV-B radiation [48]. Mitochondria play a pivotal role in plant defense against pathogens by regulating metabolism, hormone signaling, and the production of ROS and RNS, which induce PCD [47,49]. The upregulation of mitochondrial genes enhances plant defense and resilience, facilitated by ROS production, which regulates signaling [50].

The overexpression of mitochondrial genes has been observed to correlate with elevated mitochondrial activity in response to UV radiation and oxidative stress [51]. In response to stress or fluctuating light, plants may reduce photosynthesis in order to conserve energy and minimize ROS damage. This adaptive response maintains energy balance and prevents damage, indicating that plants are capable of detecting and responding protectively to environmental stimuli [47,52]. The flash treatment resulted in alterations to chloroplast and mitochondrial activity, which in turn led to shifts in energy routes. An increase in mitochondrial gene expression and a decrease in photosynthetic gene expression enhance ROS homeostasis, which may strengthen the plant’s defense and immunity mechanisms.

### 3.4. Programmed Cell Death (PCD) Events Triggered by the UV-C Flash Treatment

The findings demonstrate that UV-C radiation has induced alterations in the expression of genes associated with PCD. In addition to its function in plant development, PCD is crucial for the defense against biotic and abiotic stresses [53,54]. PCD is initiated by a number of factors, such as excess light, heat, drought, waterlogging, UV radiation, salinity, and heavy metals, and is induced by ROS [55]. The control mechanisms of PCD genetically orchestrate pathways involving protein biogenesis and modification through transcription factors, regulators, proteins, and enzymes such as peroxidases, metacaspases, and vacuolar processing enzymes.

The expression of genes such as RRTF1, DTXs, and MSS1 was altered by both 1 s and 60 s UV-C treatments (Table 5 and Supplementary File). RRTF1, an ERF109 transcription factor, regulates PCD inhibitor genes and delays PCD under conditions of high salinity by inhibiting ROS processes [56]. As a JA-responsive transcription factor, its underexpression suggests a role in activating PCD [57], thereby contributing to UV-C-stimulated plant defense mechanisms. The altered expression of PCD-related genes (Table 5), which impact ROS production and PCD [58], indicates that the flash treatment affects these genes differently due to changes in ROS activity, electron flow, and signaling.

The pattern of PCD-related gene expression suggests that the flash treatment is more efficacious than the 60 s treatment in stimulating the defense system and preparing for stress. This is due to the involvement of both ROS and PCD in resilience and stress memory mechanisms [59,60]. The flash treatment resulted in the high expression of several DEGs such as BAG6, LRX1, Bax inhibitor-1, and FTSH6 (Table 1 and Table 4). These genes play pivotal roles in PCD events in plants and are induced by biotic and abiotic stresses.

BAGs are essential for defense against pathogens and are upregulated by stress-related factors, such as salinity, heat, ABA, Et, and SA [61], which in turn trigger autophagy, signal transduction pathways, and defense mechanisms. Bax inhibitor-1 is a conserved cell death suppressor that modulates pro-survival and pro-death signals in response to various stress conditions and PCD events related to ER stress [62]. Its overexpression prevents cell death and accelerates survival mechanisms [63]. The overexpression of these genes by the flash treatment suggests it stimulates PCD events and mechanisms by which plants defend against various stresses, potentially involving the plant immune system. These mechanisms can then prepare plants treated by UV-C flash for further stress adaptation and resilience.

### 3.5. HSPs Overexpression Induced by the UV-C Flash Treatment

Of the identified DEGs, 24 HSPs and 2 HSFs (HSFA2 and HSFC1) were common to both treatments, indicating a general UV-C effect on mechanisms related to HSPs. These HSPs and HSFs operate as components of an integrated network [64]. HSFA7A and HSFA3 were exclusive to the flash treatment and HSFA6A was exclusive to the 60 s treatment (Table 5 and Supplementary File).

In the flash treatment, only five HSPs exhibited downregulation, whereas the remaining twenty-five HSPs demonstrated significant overexpression (log2FC > 3, Table 5 and Supplementary File). The expression levels of commonly overexpressed DEGs were significantly higher in the flash treatment, likely due to the rapid response required by plants exposed to the same UV-C dose in a single second. This encompasses mechanisms such as proteostasis, oxidative phosphorylation, and ROS management. Consequently, HSPs and HSFs were markedly upregulated as a protective response, playing a pivotal role in maintaining cellular integrity, preventing ROS damage, enhancing stress resistance, and ensuring proper protein folding and structure [65]. This indicates the existence of a specific expression pattern of HSP genes stimulated by the flash treatment.

HSPs are also implicated in DNA repair, interacting with proteins that are essential for maintaining genome stability [66]. This interaction orchestrates the expression of various a number of genes and mechanisms that maintain organismal health [67]. Furthermore, HSPs serve as “memory molecules”, enabling plants to respond effectively to subsequent stressors [68] due to their multifaceted roles in sensing, signaling, transcription, translation, and post-translational modifications [69]. Additionally, the excessive overexpression of HSPs can trigger priming mechanisms, thereby preparing plants for environmental changes [70].

While the overexpression of a single HSF or HSP gene has limited effects, the synergistic action of HSFs and HSPs confers a number of benefits, including homeostasis, thermotolerance, chilling/freezing tolerance, pathogen defense, heat stress resistance, and cellular stability [71]. The alteration of HSF classes A and C following UV-C exposure indicates that HSFAs are integral to transcriptional activation [72]. HSFA2 and HSFA3 interact in the same thermoregulatory pathway, regulating genes such as multiprotein bridging factor 1C (MBF1C) [73]. The observed upregulation of HSFs in response to the flash treatment provides evidence that they are responsible for the concurrent upregulation of MBF1C, which is a key regulator of HSP genes.

Furthermore, additional genes that interact with HSPs, including rotamase FKBP 1 (ROF1), BIPs, AT5G48570 (CYP57), ATG8H (autophagy 8 h), and AT2G40130 (SMAX1-like 8) [74,75], were also altered by the flash treatment. These genes are involved in thermotolerance, heat response, inhibition of virus replication, and protein modification. It can thus be posited that the flash treatment may enhance thermotolerance. Given the established role of HSPs and HSFs in plant stress memory, we hypothesize that UV-C flash treatment may prime plants for abiotic stress, particularly heat, chilling, and freezing.

### 3.6. Cell Cycle and Division and DNA Replication Blocked by the UV-C Flash Treatment

The results demonstrate that the flash treatment exerted a pronounced impact on genes implicated in the cell cycle, cell division, and DNA replication/repair. It also resulted in the downregulation of gene families, including the MCM complex, kinesin motors, ATP-binding microtubule motor proteins, and IQD proteins. These genes were not altered by the 60 s treatment, indicating a specific expression pattern for the flash treatment (Table 6 and Supplementary File).

The MCM complex is linked to uncontrolled cell division, DNA replication and repair, and cancer in humans [76]. The overexpression of MCM genes in cancerous cells serves as a biomarker for the dangerous proliferation of cells, with dysfunction impairing the processes of DNA replication and proliferation [77]. In the studied plants, the UV-C flash was observed to downregulate MCM genes, which may serve to protect cells and their genome. Furthermore, it was observed that kinesin genes involved in cell division and microtubule-based transport were downregulated [78]. Additionally, the flash treatment resulted in the downregulation of all IQD family genes (IQ67 domain), which are plant-specific calmodulin-binding proteins involved in microtubule organization, cell division, coordinated growth, and signaling pathways [79]. The expression of these genes is influenced by various environmental stresses, including cold, salinity, drought, and heat [80]. Kinesin motors are microtubule-based motor proteins and IQD proteins are responsible for the organization of microtubules [81]. In humans, the overexpression of kinesin superfamily proteins has been observed to indicate cancer cell proliferation and tumorigenesis. Conversely, gene knockdown has been demonstrated to inhibit breast cancer cell proliferation [82].

The gene network via ATRM indicates a balance between repair and growth in genes that are differentially expressed following the flash treatment. Genes associated with the cell cycle, cell division, and DNA replication/repair (e.g., CDCs, CDKs, HMGs, PCNA1, and PRL) exhibited downregulation, accompanied by the upregulation of DNA repair genes, including ETG1, CDC6, and EDA35. In comparison to the 60 s treatment, the flash treatment resulted in alterations to twenty cyclin-related genes, in contrast to the six observed in the latter. ETG1, CDC6, HMGs, CDKs, and EDA35 are involved in maintaining genome stability and regulating DNA replication checkpoints [83,84]. The downregulation of CYCs is responsible for orchestrating the cell cycle, while the upregulation of other genes serves to stabilize the genome.

The results of this study indicate that cell cycle arrest may represent a survival and protective strategy for plants subjected to a flash treatment. It is postulated that a UV-C flash may impede DNA replication and cell division, thereby facilitating the repair of damaged DNA before new copies are made, and induce a halt in the cell cycle, thus protecting the cell. The downregulation of kinesin and IQD genes indicates a temporary cessation of cell cycle activity [85], thereby allowing plants to prioritize DNA repair, the synthesis of protective compounds, or the activation of defense mechanisms. The cessation of the cell cycle allows for an extended period during which DNA repair mechanisms can address the effects of UV-C radiation, thereby facilitating the synthesis of stronger cell walls and more effective defense signaling. Cell cycle arrest can indicate the presence of stress via the production of ROS or DNA damage, which in turn triggers the activation of defense pathways and the synthesis of antioxidants [86]. Cell cycle arrest is of great importance for programmed cell death (PCD) during immunity, as it enables the maintenance of genomic integrity, the elimination of damaged cells, and the assurance of tissue health [87,88]. The connection between arrested cell cycle progression and plant immunity is mediated by ROS and endoreplication regulation, with cell cycle components serving as regulators of immunity [89].

The application of a flash treatment to plants resulted in distinctive cellular alterations in gene expression which were associated with cell cycle arrest, DNA repair, transcriptional regulation, and mitochondrial hyperactivity. These observations suggest a potential correlation between flash treatment and plant health and immunity. The flash-treated plants enter a transient state associated with protection against the effects of UV-C light, which is characterized by cell cycle arrest, gene expression regulation, and DNA repair. It is probable that this state also prepares the plants against potential pathogen attacks and adverse environmental conditions. Subsequently, these expression trends mirror those observed in pathogen-attacked plants, thereby supporting the hypothesis of a trade-off between immunity and cell cycle arrest [89,90]. The downregulation of these genes by flash treatment suggests roles in cellular processes, epigenetic regulation, signal transduction, cytoskeletal organization, and gene expression regulation, thereby contributing to the establishment of stress memory mechanisms [91]. The coordinated regulation of these genes suggests that flash treatment may enhance plant immunity via pathways that have not been previously reported and that are involved in stress adaptation in plants.

### 3.7. Cell Wall Remodeling, Integrity Maintenance and PatternTriggered Immunity Prompted by the UV-C Flash Treatment

The results demonstrate a notable discrepancy in the quantity and manifestation of cell wall-associated genes between the experimental groups (Supplementary File). The flash treatment affected a greater number of genes related to cell wall integrity and remodeling than the 60 s treatment, with a ratio of approximately twofold. For example, fourteen polygalacturonase (PG) genes exhibited differential expression in plants treated with flash, compared to seven in the 60 s treatment. Eight DEGs from the AGP gene family were identified in the flash treatment, in comparison to two in the 60 s treatment. The flash treatment resulted in the downregulation of six pectate lyase genes, while no specific pectate lyase DEGs were observed in the 60 s treatment. The flash treatment resulted in the specific upregulation of nine hydroxyproline-rich glycoproteins (HRGPs), while one was downregulated. In contrast, the 60 s treatment led to the specific downregulation of two HRGPs. Genes encoding extracellular and transmembrane proteins, as well as cell wall receptors and sensors, exhibited high expression levels (more than 10 times) in the flash treatment. This included LRX1, AGP2, LLG3, PELPK2, PGA3, FLA3, ADPG1, QRT2, and HRGP. In the 60 s treatment, only one cell wall-related gene, AT1G78400 (a glycoside hydrolase family 28 protein/polygalacturonase), exhibited high expression levels. Some DEGs have been found to correspond to enzymes that degrade carbohydrate polymers, such as cellulose and pectin. The degradation of these polymers produces signaling molecules, including oligogalacturonides [92], which can trigger plant defense responses.

LRX1 (leucine-rich repeat/extensin 1) is an extracellular component that plays a pivotal role in maintaining cell wall integrity, influencing root growth, and functioning as a cell wall receptor that detects environmental cues for signaling. Extensin networks serve to impede pathogen invasion by modifying the cell wall [93]. LRXs have been demonstrated to interact with cell wall components [94]. LLGs are integral membrane-associated proteins that regulate a multitude of cellular processes, including cell–cell communication, growth, reproduction, immunity, and stress responses [95]. LLG2/3 constitute a receptor–coreceptor complex that detects RALF4/9 peptide signals, thereby modulating cell wall integrity [96]. Polygalacturonases (PGs) are enzymes that depolymerize pectin in the cell wall, contributing to the overall architecture and pathogen resistance, including against bacterial blight. The expression of pectate lyase genes is repressed by the flash treatment, which prevents the degradation of the cell wall and increases its rigidity, enhancing stability and resistance.

Receptors and elicitors that are localized to the cell wall, including the ectopic expression of ADPG1 in the xylem, which is normally expressed during anther and silique dehiscence, result in the expression of PR genes and the release of elicitors. ADPG1 and QRT2 function synergistically to degrade pectin and release PR elicitors [97,98]. AGPs contribute to functional diversity and may function as cell surface adhesion proteins, coordinating signaling with growth and development [99]. AGPs (extracellular hydroxyproline-rich glycoproteins) are involved in maintaining cell wall integrity, regulating growth, and mediating signaling in plant–pathogen interactions [100]. The HRGP family plays a pivotal role in growth, development [101] and defense [102]. HRGPs serve as sensors and transducers of environmental signals, thereby initiating signaling pathways. The overexpression of HRGP genes by the flash treatment indicates that it enhances cell wall strength without impeding growth, thereby balancing defense and growth through enhanced cross-linking. The observed cell wall gene expression in plants treated with UV-C flashes represents a specific response that is involved in maintaining cell wall integrity, preventing vulnerability to UV-C flashes or pathogens, and maintaining signaling functions.

### 3.8. Ordered View of the Effect of the UV-C Flash Treatment

**Figure 11:**
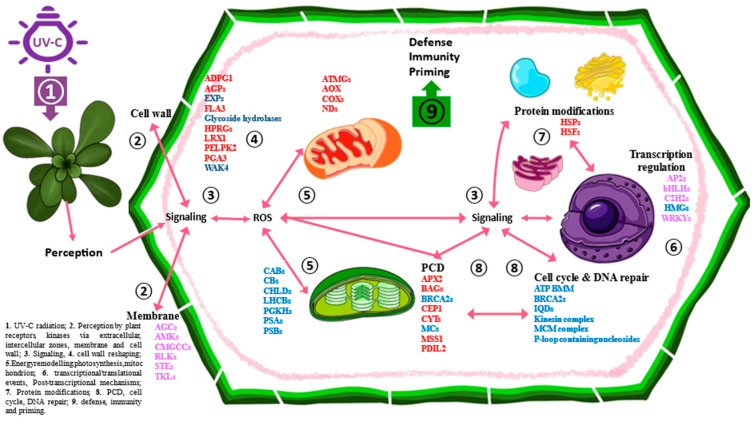
Hypothetical vision of plant responses to the flash treatment. 1. UV-C light flashes. 2. Perception: Plants receive UV-C light via receptors, proteins, and molecules in the membrane, cell wall, and extracellular and intercellular zones. 3. Signaling initiation: interaction of receptors, cell wall, and membrane proteins begins signaling. 4. Cell wall modification: enzymes and proteins modify cell wall integrity, producing signaling molecules from compounds like pectin and cellulose. 5. ROS production: chloroplasts and mitochondria produce and scavenge ROS, which are also used as signaling molecules. Electron transport flow and energy remodeling occur. 6. Transcription regulation: nucleus, nucleolus, and other organelles coordinate interactions between genes and proteins, notably in mitochondria and chloroplasts. Expression of ATCG and ATMG is regulated by cell genes. 7. Protein modifications: overexpression of HSPs, HSFs, and related genes indicates protein modifications involved in signaling and transcriptional regulation. 8. PCD: PCD genes are expressed, collaborating with ROS, cell cycle, DNA repair, and signaling activities. 9. Defense activation: Differential gene expression activates defense, immunity, and priming systems. Genes in blue are overexpressed, red are underexpressed, and pink show differential expression patterns (both over- and underexpression).

Our findings provide insights into how UV-C flashes elicit plant responses, such as defenses, as shown in previous studies [12,14,17,18]. The response to the flash treatment commences with the reception of UV-C light, which is linked to oxidative/photooxidative stress, ROS management, alterations of cell wall and membrane integrity, and light penetration in cells and organelles. These events serve to initiate signaling responses at the chloroplast, mitochondrial, and nuclear levels, which have been observed to fulfill a variety of functions, including acclimation to environmental stress and the defense against pathogens (Figure 11).

The cell nucleus and nucleolus are stimulated by signaling elements that orchestrate a range of processes, including transcription, post-transcriptional events, and post-translational processes. Transcription factors and regulators participate in downstream mechanisms and pathways associated with responses to the UV-C flash treatment, which in turn contribute to enhanced immunity and tolerance against abiotic stress. The aforementioned processes of cell cycle arrest and DNA repair serve as acclimation strategies that protect cells.

The flash treatment demonstrated the coordinated expression of numerous gene families involved in diverse biological processes, including the cell cycle, DNA repair and replication, transcriptional and post-transcriptional regulation, mitochondrial and chloroplast functions, cell wall and membrane integrity, and protein modifications. The expression of these gene families was not affected by the 60 s treatment. This suggests that the plant responses to the two treatments are fundamentally disparate. The disparate gene expression patterns and treatment-specific DEGs suggest that the flash treatment exerts a priming effect that can ultimately be employed by plants against pathogens and stressful conditions, while the 60 s treatment primarily elicits a more direct protective response. Moreover, specific responses to the flash treatment were observed in the gene expression patterns of certain genes. For instance, several genes that were altered by the flash treatment, which had previously been reported to affect pollen tube growth, are involved in the defense response when plants are exposed to environmental stresses. In conclusion, we propose the UV-C flash treatment as a novel approach for plant priming. This technique has been observed to stimulate the expression of multifunctional genes involved in growth, development, signaling, and transcriptional regulation in ways that differ from those observed in traditional priming, defense, and immunity mechanisms. The application of UV-C light flashes to plants results in a distinctive response to environmental stimuli, which in turn reveals novel pathways and mechanisms. These pathways involve genes whose functions were previously unidentified in the context of plant priming and resilience, yet they play a pivotal role. Further research is necessary to elucidate the mechanisms by which these genes altered by flashes of UV-C light orchestrate and trigger plant responses to stress adaptation.

## 4. Materials and Methods

### 4.1. Characteristics (Feature) of UV-C Lamp

UV-C light was generated by a system of ten UV-C amalgam lamps (OSRAM PURITEC, HNS L, 95 W, GFT95DL/2G11/SE/OF, 254 nm) housed in a 60 × 60 cm aluminum frame. This apparatus was specifically designed to provide 1 s flashes under greenhouse and field conditions (UV Boosting, Boulogne-Billancourt, France) [17]. The spectrum was measured using a UV sensor (OSI UV-20 TO-8 photodiode) and a prominent peak at 254 nm was confirmed. The energy perceived by plants was contingent upon the distance between the UV-C source and the plants themselves. The light energy was quantified using a radiometer RM12 (Opsytec Dr. Gröbel GmbH, Ettlingen, Germany). Based on preliminary experiments, a dose of 200 J m^−2^ delivered in one second was found to be effective in stimulating the defenses of *A. thaliana* L. plants without any negative phenotypic effect on plant growth and appearance (Supplementary Figure S1).

### 4.2. Plant Material and UV-C Treatments

*A. thaliana* L. (Cv col0) plants were sown on ½ MS medium plates supplemented with 1% agar and maintained for two days at 4 °C in the dark. Subsequently, the plates were transferred to a climatic chamber and after five days the plantlets were transferred to 7 × 7 cm pots filled with soil (with a single plantlet in each pot). Then, the plants were grown in a phytotron for a period of five weeks under constant conditions, including a photosynthetically active radiation (PAR) of 50 μmol photons m^−2^ s^−1^, photoperiod of 12 h/12 h with an average day/night temperature of 21 °C/20 °C and 70% relative humidity. The five-week-old t plants were subjected to a single dose of 200 J m^−2^ UV-C light, delivered either as a 1 s (flash treatment) or as a 60 s exposure (60 s treatment) or were left untreated and served as the control group. The treated plants were then compared with the control group. Five plants per treatment were used for analyses. The plants were subjected to three distinct applications of UV-C radiation, with a one-week interval between each successive application. The treated plants were returned to the phytotron after each treatment. Leaves from UV-C-treated and untreated plants were harvested four hours after the final UV-C treatment and immediately frozen in liquid nitrogen. They were then stored at −80 °C. The frozen samples were crushed and pulverized in a sterile mortar with liquid nitrogen and stored at −80 °C until RNA extraction.

### 4.3. RNA Sequencing

Total RNA was extracted from 100 mg of ground leaves using the RNeasy Plant Kit (QIAGEN France S.A.S, Courtaboeuf, France) following the manufacturer’s instructions. The quantity of the RNA was determined using a Qbit 4 Fluorometer (Thermo Scientific, Wilmington, NC, USA) and the RNA quality was assessed using a Fragment Analyzer (Agilent Technologies, Santa Clara, CA, USA). All RNA samples exhibited an RNA Integrity Number (RIN) score of seven or above. Library preparation and RNA-seq were performed by Beijing Genomics Institute (BGI) Tech Solution (Hong Kong, China) using a DNBSEQ platform.

### 4.4. Differential Gene Expression, GO and KEGG Analysis

RNA-seq data were analyzed using Dr Tom tools (https://biosys.bgi.com/help/en/ (accessed on 18 December 2024)), an online software program developed by BGI Genomics, (Shenzhen, China). The reads were previously mapped to the Arabidopsis TAIR10 reference genome.

To identify differentially expressed genes (DEGs) between UV-C-exposed and control plants, the DESeq2 tool was used [103]. To filter significant DEGs, an adjusted *p*-value of ≤0.05 (FDR) and |log2 (fold change)| > 1 were defined.

Dr Tom tools was used to perform functional classification of the differentially expressed protein-coding genes through Gene Ontology (GO) enrichment and KEGG enrichment. This analysis helps highlight biological processes, molecular functions, and cellular components that are significantly associated with the observed gene expression changes.

### 4.5. Functional Pathway and Annotation Analysis

The DEGs were analyzed using MapMan software Version 3.6.0RC1 (https://mapman.gabipd.org/mapman (accessed on 18 December 2024)) [104] to gain insight into their biological functions and pathways. This software allowed researchers to assign DEGs to desired pathways, for which the gene IDs of the DEGs were mapped to genes in the TAIR10 database (Arabidopsis Information Resource), enabling the exploration of specific pathways relevant to the study. Researchers also analyzed gene families, as MapMan also facilitates the examination of the gene families to which the identified DEGs belong, providing further context for their potential roles and interactions. To investigate the functional roles of the DEGs, the results of the Gene Ontology (GO) analysis for biological processes and cellular components were integrated with those of the Kyoto Encyclopedia of Genes and Genomes (KEGG, https://www.genome.jp/kegg/ (accessed on 18 December 2024)) pathways [105], which were then subjected to further examination. This allowed to connect the DEGs to specific biological functions and processes within the cell and reactions. The DEGs and their respective expression values were submitted to KEGG database and the desired maps with colored DEGs assigned to the maps were employed for data interpretation related to the desired gene families.

To gain deeper functional insights, The Database for Annotation, Visualization, and Integrated Discovery (DAVID) bioinformatics resources based on DAVID Knowledgebase v2024q2 (https://davidbioinformatics.nih.gov/ (accessed on 18 December 2024)) [106,107] were utilized to identify and assess the overrepresentation of specific GO terms among the DEGs. DAVID’s functional annotation tools were used to enrich the data with information drawn from a range of publicly accessible resources. This process provided supplementary insights into the potential roles and biological contexts of the DEGs.

### 4.6. Classification of Transcription Factors and Regulators

To identify and categorize potential transcriptional regulators involved in the observed differential gene expression, PlantTFcat (An online plant transcription factor and transcriptional regulator categorization and analysis tool) was employed. It is a web-based tool (https://www.zhaolab.org/PlantTFcat/ (accessed on 18 December 2024)) specifically designed for analyzing plant transcription factors and regulatory elements [108]. This approach offers several advantages over traditional methods, including high-throughput analysis, comprehensive coverage, and family-level categorization into transcription factor (TF), transcription regulator (TR), and chromatin remodeling factor (CR) motifs. Specifically, PlantTFcat analyzes the input sequences, searching for the distinctive motifs characteristic of each TF/TR/CR family within its comprehensive database. PlantTFcat assigns sequences to specific families based on their domain composition, thereby providing valuable insights into potential regulatory mechanisms and functional roles.

The Arabidopsis Transcriptional Regulatory Map (ATRM) [109], a tool integrated within PlantTFDB Version 4.0 (https://planttfdb.gao-lab.org/ (accessed on 18 December 2024)) [110], was employed to map the regulatory and transcriptional interactions between the identified DEGs. This analysis facilitated the elucidation of the relationships between specific TFs and their target DEGs within a comprehensive regulatory network.

### 4.7. Validation of RNA-Seq

To corroborate the data obtained from RNA-seq analysis, the gene expression of five selected genes, identified as DEGs, was validated through RT-qPCR. A total of 100 ng of total RNA was used for cDNA synthesis, employing an oligo-(dT)18 anchor primer and the Reverse Transcriptase Core Kit (Eurogentec, Angers, France) according to the manufacturer’s instructions. RT-qPCR amplification was conducted with the Takyon No Rox SYBR Core Kit dTTP blue (Eurogentec) on a Mastercycler ep realplex cycler (Eppendorf, Hamburg, Germany). PCRs were performed in a 96-well plate, using a three-fold cDNA dilution in triplicate as template, with 200 nM forward and reverse primers. The specific primers of targeted genes are listed in the supplemental data. Actin (ACT) and Ubiquitin 9 (UBQ9) genes were used as an internal control. The relative expression levels of the target genes were calculated as fold changes in expression using the 2^−ΔΔCq^ method [111], with the mean Cq values corresponding to the mean of the five biological replicates. The results were transformed using the log2 function.

## 5. Conclusions

The original observations by Aarrouf and Urban demonstrated a distinct superiority of the UV-C flash treatment over 60 s exposures for the stimulation of plant defense mechanisms [14]. The results of our study do not support the hypothesis that the UV-C flash treatment is superior. Conversely, conventional UV-C light exposures exert a more straightforward influence on plant defense. Although some common responses were observed between the flash and conventional treatments, our data demonstrate that plants undergo more profound changes with the UV-C flash, including the remodeling of energy flux, cell protection mechanisms, and cell wall integrity and the upregulation of genes involved in oxidative stress and epigenetic processes. The flash treatment induces distinctive features, including the upregulation of PCD genes, cell cycle arrest, and DNA repair, which collectively suggest an enhanced defense potential. However, this potential is less direct than that observed with conventional exposures. The upregulation of HSP genes indicates that the flash treatment has a priming effect on immunity, potentially stimulating thermotolerance and other abiotic stress tolerances. The flash treatment activates the mitochondria, chloroplasts, and nucleus to execute these effects. It is postulated that the priming effect of the flash treatment is more extensive and potentially more efficacious over the long term, necessitating further evidence to accurately orient defense responses. The results of this study indicate that UV-C flashes have the potential to stimulate crop thermotolerance, which is an important issue in the context of climate change.

From an agronomic standpoint, it is recommended that three repeated UV-C flashes be administered well in advance of stress conditions or pathogen development. Nevertheless, the deployment of this technology is constrained by some factors, including the accessibility of UV-C lamps, the scalability of the UV-C flash approach in field conditions, the determination of the optimal application time to stimulate plant defense and immunity in different species, and the assessment of potential effects on other living organisms following UV-C exposure. Further trials are required to investigate these hypotheses and address the remaining questions regarding repeated treatments, UV-C light perception, stimulating immunity, and priming.

## Figures and Tables

**Table 1:** The DEGs associated with chloroplast and ATCG genes. The ATCG genes are encoded by the chloroplast genome. The DEGs related to NAD, RNA polymerase, PSI, PSII, electron transfer, and ATP are among them. The complete list of chloroplast and photosynthetic DEGs can be found in the Supplementary File.

GeneID	Gene Symbol	Gene Name	Log2FC (Flash Treatment)	FDR (Flash Treatment)
ATCG00020	PSBA	Photosystem II reaction center protein A	1.58	8.78 × 10^−11^
ATCG00040	MATK	Maturase K	2.10	8.42 × 10^−7^
ATCG00130	ATPF	ATPase, F0 complex, subunit B/B’, bacterial/chloroplast	0.97	5.36 × 10^−3^
ATCG00140	ATPH	ATP synthase subunit C family protein	0.84	1.88 × 10^−1^
ATCG00170	RPOC2	DNA-directed RNA polymerase family protein	−1.18	3.61 × 10^−2^
ATCG00180	RPOC1	DNA-directed RNA polymerase family protein	−1.12	9.33 × 10^−3^
ATCG00190	RPOB	RNA polymerase subunit beta	−1.07	8.05 × 10^−4^
ATCG00270	PSBD	Photosystem II reaction center protein D	1.69	4.68 × 10^−10^
ATCG00280	PSBC	Photosystem II reaction center protein C	1.37	2.25 × 10^−13^
ATCG00340	PSAB	Photosystem I, PsaA/PsaB protein	0.96	1.95 × 10^−6^
ATCG00350	PSAA	Photosystem I, PsaA/PsaB protein	0.84	2.85 × 10^−3^
ATCG00490	RBCL	Ribulose-bisphosphate carboxylases	0.90	3.98 × 10^−4^
ATCG00500	ACCD	Acetyl-CoA carboxylase carboxyl transferase subunit beta	1.72	2.71 × 10^−2^
ATCG00720	PETB	Photosynthetic electron transfer B	1.65	7.75 × 10^−11^
ATCG00730	PETD	Photosynthetic electron transfer D	1.22	9.13 × 10^−7^
ATCG00790	RPL16	Ribosomal protein L16	0.01	9.90 × 10^−1^
ATCG01050	NDHD	NADH-Ubiquinone/plastoquinone (complex I) protein	1.83	3.57 × 10^−11^
ATCG01070	NDHE	NADH-ubiquinone/plastoquinone oxidoreductase chain 4 L	2.27	1.36 × 10^−1^
ATCG01080	NDHG	NADH: ubiquinone/plastoquinone oxidoreductase, chain 6	2.62	6.47 × 10^−5^
ATCG01100	NDHA	NADH dehydrogenase family protein	1.44	4.29 × 10^−3^
AT5G15250	FTSH6	FTSH protease 6	5.07	1.39 × 10^−14^

**Table 2:** The DEGs related to mitochondrial processes encoded by the nuclear genome. The expression of these genes was predominantly elevated in the flash treatment, whereas the 60 s treatment resulted in minimal alterations in their expression levels. They are involved in processes including mitochondrial electron transport, RNA regulation and transcription, protein synthesis, modification, translocation, and folding, and metabolite biosynthesis and play roles in stress responses and adaptation and transport across the mitochondrial membrane. They belong to protein categories including enzymes, chaperons, transporters, and binding proteins. The genes are classified according to their annotation, function, and the results of the following analyses: GO enrichment, MapMan, DAVID, and KEGG.

GeneID	Gene Symbol	Gene Name	Log2FC (Flash Treatment)	FDR (Flash Treatment)
AT1G02370	AT1G02370	Tetratricopeptide repeat (TPR)-like superfamily protein	1.42	7.57 × 10^−13^
AT1G12060	BAG5	BCL-2-associated athanogene 5	1.02	2.65 × 10^−2^
AT1G15480	AT1G15480	Tetratricopeptide repeat (TPR)-like superfamily protein	1.04	7.55 × 10^−6^
AT1G15870	AT1G15870	Mitochondrial glycoprotein family protein	1.14	1.29 × 10^−5^
AT1G18320	AT1G18320	Mitochondrial import inner membrane translocase subunit Tim17/Tim22/Tim23 family protein	1.34	3.25 × 10^−2^
AT1G23800	ALDH2B7	Aldehyde dehydrogenase 2B7	1.59	1.06 × 10^−4^
AT1G28210	ATJ1	DNAJ heat shock family protein	1.40	1.69 × 10^−15^
AT1G30370	DLAH	DAD1-like acylhydrolase	−1.92	6.53 × 10^−3^
AT1G45332	AT1G45332	Translation elongation factor EFG/EF2 protein	1.03	2.96 × 10^−14^
AT1G52160	TRZ3	tRNAse Z3	1.20	4.23 × 10^−16^
AT1G53600	AT1G53600	Tetratricopeptide repeat (TPR)-like superfamily protein	1.02	7.48 × 10^−3^
AT1G61990	AT1G61990	Mitochondrial transcription termination factor family protein	1.28	6.75 × 10^−12^
AT1G62010	AT1G62010	Mitochondrial transcription termination factor family protein	1.08	2.21 × 10^−7^
AT1G62110	AT1G62110	Mitochondrial transcription termination factor family protein	1.10	4.28 × 10^−4^
AT1G62380	ACO2	ACC oxidase 2	1.21	6.20 × 10^−9^
AT1G62720	NG1	Novel gene 1	1.49	2.53 × 10^−5^
AT1G65080	AT1G65080	Membrane insertion protein, OxaA/YidC with tetratricopeptide repeat domain	1.05	2.09 × 10^−9^
AT1G68990	MGP3	Male gametophyte defective 3	1.15	1.04 × 10^−20^
AT1G78930	AT1G78930	Mitochondrial transcription termination factor family protein	1.81	2.86 × 10^−12^
AT1G80150	AT1G80150	Tetratricopeptide repeat (TPR)-like superfamily protein	1.31	3.21 × 10^−9^
AT2G07707	AT2G07707	Plant mitochondrial ATPase, F0 complex, subunit 8 protein	2.43	1.67 × 10^−7^
AT2G17270	PHT3;3	Phosphate transporter 3;3	1.01	3.44 × 10^−6^
AT2G25140	CLPB4	Casein lytic proteinase B4	1.95	8.09 × 10^−12^
AT2G28050	AT2G28050	Pentatricopeptide repeat (PPR) superfamily protein	−1.35	3.49 × 10^−3^
AT2G34620	AT2G34620	Mitochondrial transcription termination factor family protein	−1.33	8.76 × 10^−3^
AT2G38400	AGT3	Alanine: glyoxylate aminotransferase 3	1.57	1.64 × 10^−33^
AT2G39120	WTF9	What’s this factor 9	1.48	3.79 × 10^−5^
AT2G39230	LOJ	Lateral organ junction	1.41	3.57 × 10^−8^
AT2G39725	AT2G39725	LYR family of Fe/S cluster biogenesis protein	1.33	2.88 × 10^−8^
AT2G40240	AT2G40240	Tetratricopeptide repeat (TPR)-like superfamily protein	1.09	1.05 × 10^−8^
AT2G41670	AT2G41670	P-loop containing nucleoside triphosphate hydrolases superfamily protein	1.40	3.98 × 10^−14^
AT2G46110	KPHMT1	Ketopantoate hydroxymethyltransferase 1	−1.18	2.31 × 10^−8^
AT3G07770	Hsp89.1	Heat shock protein 89.1	1.16	4.65 × 10^−12^
AT3G10680	AT3G10680	HSP20-like chaperones superfamily protein	−1.27	3.88 × 10^−2^
AT3G13880	AT3G13880	Tetratricopeptide repeat (TPR)-like superfamily protein	1.47	1.16 × 10^−5^
AT3G15000	RIP1	RNA-editing factor interacting protein 1	1.06	6.31 × 10^−14^
AT3G17465	RPL3P	Ribosomal protein L3 plastid	1.09	2.68 × 10^−8^
AT3G17611	RBL14	RHOMBOID-like protein 14	1.66	5.56 × 10^−7^
AT3G19440	AT3G19440	Pseudouridine synthase family protein	1.40	5.24 × 10^−8^
AT3G27380	SDH2−1	Succinate dehydrogenase 2-1	1.31	9.63 × 10^−8^
AT3G30775	ERD5	Early responsive to dehydration 5	1.92	8.67 × 10^−7^
AT3G45300	IVD	Isovaleryl-CoA-dehydrogenase	1.04	1.06 × 10^−8^
AT3G46950	AT3G46950	Mitochondrial transcription termination factor family protein	1.11	8.66 × 10^−5^
AT3G48250	BIR6	Buthionine sulfoximine-insensitive roots 6	1.13	2.92 × 10^−7^
AT3G49240	emb1796	Embryo defective 1796	1.45	3.69 × 10^−11^
AT3G51790	G1	Transmembrane protein G1P-related 1	1.29	2.67 × 10^−6^
AT4G04790	AT4G04790	Tetratricopeptide repeat (TPR)-like superfamily protein	1.01	1.05 × 10^−6^
AT4G11060	MTSSB	Mitochondrially targeted single-stranded DNA-binding protein	1.22	2.37 × 10^−5^
AT4G13850	GR-RBP2	Glycine-rich RNA-binding protein 2	1.18	7.85 × 10^−14^
AT4G20020	MORF1	Multiple organellar RNA editing factor 1	1.08	2.64 × 10^−6^
AT4G26780	AR192	Co-chaperone GrpE family protein	1.32	1.38 × 10^−6^
AT4G28390	AAC3	ADP/ATP carrier 3	1.06	1.88 × 10^−6^
AT4G32210	SDH3-2	Succinate dehydrogenase 3-2	1.26	3.41 × 10^−2^
AT4G36680	AT4G36680	Tetratricopeptide repeat (TPR)-like superfamily protein	1.32	1.11 × 10^−13^
AT4G37930	SHM1	Serine transhydroxymethyltransferase 1	−1.13	6.17 × 10^−8^
AT5G01340	mSFC1	Mitochondrial succinate-fumarate carrier 1	2.22	7.61 × 10^−9^
AT5G07440	GDH2	Glutamate dehydrogenase 2	1.25	5.10 × 10^−5^
AT5G08305	AT5G08305	Pentatricopeptide repeat (PPR) superfamily protein	1.09	6.98 × 10^−3^
AT5G09590	MTHSC70-2	Mitochondrial HSO70 2	1.97	5.18 × 10^−9^
AT5G14180	MPL1	Myzus persicae-induced lipase 1	1.73	3.24 × 10^−7^
AT5G15090	VDAC3	Voltage dependent anion channel 3	1.17	1.98 × 10^−6^
AT5G15640	AT5G15640	Mitochondrial substrate carrier family protein	1.40	1.53 × 10^−10^
AT5G15700	AT5G15700	DNA/RNA polymerases superfamily protein	1.29	3.99 × 10^−21^
AT5G23300	PYRD	Pyrimidine d	1.04	1.50 × 10^−7^
AT5G23930	AT5G23930	Mitochondrial transcription termination factor family protein	1.07	3.80 × 10^−3^
AT5G26860	LON1	Lon protease 1	1.76	1.10 × 10^−37^
AT5G27395	AT5G27395	Mitochondrial inner membrane translocase complex, subunit Tim44-related protein	1.05	2.42 × 10^−8^
AT5G44780	MORF4	Multiple organellar RNA editing factor 4	1.46	7.06 × 10^−11^
AT5G46920	AT5G46920	Intron maturase, type II family protein	1.23	3.26 × 10^−19^
AT5G51740	AT5G51740	Peptidase family M48 family protein	1.05	1.63 × 10^−8^
AT5G56090	COX15	Cytochrome c oxidase 15	1.50	7.12 × 10^−39^
AT5G61030	GR-RBP3	glycine-rich RNA-binding protein 3	1.55	2.15 × 10^−9^
AT5G61810	APC1	ATP/phosphate carrier 1	1.20	1.32 × 10^−7^
AT5G61880	AT5G61880	Protein Transporter, Pam16	1.32	9.99 × 10^−6^
AT5G66760	SDH1-1	Succinate dehydrogenase 1-1	1.10	1.15 × 10^−9^
AT1G32350	AOX1D	Alternative oxidase 1D	1.75	9.5 × 10^−4^
AT3G22370	AOX1A	Alternative oxidase 1A	2.23	4.63 × 10^−29^

**Table 3:** The DEGs assigned to ATMG genes. These genes are encoded by the mitochondrial genome. The expression of these genes was predominantly elevated in the flash treatment, whereas the 60 s treatment had minimal impact on their expression levels. These genes are primarily involved in ATP production, NADH oxidation, electron and molecule transport, and protein biosynthesis. The majority of these genes are involved in ATPase, oxidase, and ribosomal protein functions. The genes are classified according to their annotation, function, and the results of the following analyses: GO enrichment, MapMan, DAVID, and KEGG.

GeneID	Gene Symbol	Gene Name	Log2FC (Flash Treatment)	FDR (Flash Treatment)
ATMG00060	NAD5C	NADH dehydrogenase subunit 5C	2.92	9.80 × 10^−8^
ATMG00070	NAD9	NADH dehydrogenase subunit 9	1.91	2.88 × 10^−6^
ATMG00080	RPL16	Ribosomal protein L16	2.88	1.43 × 10^−7^
ATMG00090	RPS3	RESISTANCE TO PSEUDOMONAS SYRINGAE 3	3.60	5.00 × 10^−19^
ATMG00110	ABCI2	ATP-binding cassette I2	2.52	1.36 × 10^−2^
ATMG00160	COX2	Cytochrome oxidase 2	3.59	2.85 × 10^−12^
ATMG00170	ORF139A	Transmembrane protein	4.38	2.88 × 10^−3^
ATMG00180	CCB452	Cytochrome c biogenesis 452	2.68	1.40 × 10^−8^
ATMG00270	NAD6	NADH dehydrogenase 6	4.98	2.23 × 10^−10^
ATMG00285	NAD2A	NADH dehydrogenase 2A	3.48	5.15 × 10^−10^
ATMG00290	RPS4	Mitochondrial ribosomal protein S4	3.83	6.40 × 10^−3^
ATMG00410	ATP6-1	ATPase subunit 6-1	2.71	1.57 × 10^−8^
ATMG00480	ORFB	ATPase subunit 6-1	2.43	1.67 × 10^−7^
ATMG00510	NAD7	NADH dehydrogenase subunit 7	2.00	1.23 × 10^−5^
ATMG00513	NAD5A	NADH dehydrogenase 5A	3.76	2.09 × 10^−12^
ATMG00516	NAD1C	NADH dehydrogenase 1C	2.84	3.76 × 10^−2^
ATMG00520	MATR	Intron maturase, type II family protein	1.90	1.89 × 10^−5^
ATMG00560	RPL2	Nucleic acid-binding, OB-fold-like protein	4.66	1.38 × 10^−7^
ATMG00570	ORFX	Sec-independent periplasmic protein translocase	2.39	4.36 × 10^−4^
ATMG00580	NAD4	NADH dehydrogenase subunit 4	2.13	1.71 × 10^−8^
ATMG00620	ORF139B	Transmembrane protein	4.38	2.88 × 10^−3^
ATMG00630	ORF110B	Hypothetical protein	4.03	3.71 × 10^−3^
ATMG00640	ORF25	Hydrogen ion transporting ATP synthases, rotational mechanism; zinc ion binding	2.14	6.31 × 10^−7^
ATMG00650	NAD4L	NADH dehydrogenase subunit 4L	2.04	6.60 × 10^−3^
ATMG00660	ORF149	Hypothetical protein	1.24	6.01 × 10^−3^
ATMG00690	ORF240A	FO-ATPase subunit	3.23	8.15 × 10^−11^
ATMG00730	COX3	Cytochrome c oxidase subunit 3	2.87	1.75 × 10^−6^
ATMG00830	CCB382	Cytochrome c biogenesis 382	1.86	4.35 × 10^−4^
ATMG00900	ABCI3	ATP-binding cassette I3	5.00	4.50 × 10^−4^
ATMG00940	ORF164	DNA binding	3.30	7.88 × 10^−3^
ATMG00960	CCB203	Cytochrome c assembly protein	2.69	9.22 × 10^−3^
ATMG00980	RPSL2	Ribosomal protein S12/S23 family protein	1.28	8.67 × 10^−3^
ATMG01000	ORF114	Hypothetical protein	1.54	1.53 × 10^−2^
ATMG01020	ORF153B	Transmembrane protein	1.97	1.17 × 10^−2^
ATMG01080	ATP9	Mitochondrial F0-ATPase subunit 9	1.24	2.86 × 10^−4^
ATMG01120	NAD1B	NADH dehydrogenase 1B	3.49	6.70 × 10^−3^
ATMG01130	ORF106F	Hypothetical protein	2.91	2.68 × 10^−4^
ATMG01190	ATP1	ATP synthase subunit 1	3.74	9.00 × 10^−16^
ATMG01200	ORF294	ATPase, F1 complex, alpha subunit	3.13	1.12 × 10^−7^
ATMG01220	ORF113	Hypothetical protein	1.57	3.44 × 10^−3^
ATMG01280	ORF291	Cytochrome c oxidase subunit II-like, transmembrane domain	2.25	1.17 × 10^−4^
ATMG01320	NAD2B	NADH dehydrogenase 2B	2.97	1.62 × 10^−5^
ATMG01350	ORF145C	Transmembrane protein	3.26	3.92 × 10^−2^
ATMG01360	COX1	Cytochrome oxidase	3.62	2.68 × 10^−18^
ATMG01370	ORF111D	Transmembrane protein	1.68	1.76 × 10^−2^

**Table 4:** The DEG related to PCD. These genes can be involved in different mechanisms that result in PCD processes. The genes are classified according to their annotation, function, and the results of the following analyses: GO enrichment, MapMan, DAVID, and KEGG.

GeneID	Gene Symbol	Gene Name	Log2FC (Flash Treatment)	FDR (Flash Treatment)
AT1G03070	AT1G03070	Bax inhibitor-1 family protein	5.17	1.06 × 10^−19^
AT1G04980	PDIL2-2	PDI-like 2-2	1.41	9.11 × 10^−16^
AT1G12060	BAG5	BCL-2-associated athanogene 5	1.02	2.65 × 10^−2^
AT1G16420	MC8	Metacaspase 8	−1.14	4.11 × 10^−2^
AT1G55490	CPN60B	Chaperonin 60 beta	−1.10	4.27 × 10^−3^
AT1G72860	AT1G72860	Disease resistance protein (TIR-NBS-LRR class) family	−1.02	3.70 × 10^−2^
AT1G72930	TIR	Toll/interleukin-1 receptor-like	−1.21	3.58 × 10^−9^
AT1G73260	KTI1	Kunitz trypsin inhibitor 1	1.05	1.25 × 10^−1^
AT2G07727	AT2G07727	Di-haem cytochrome, transmembrane; Cytochrome b/b6, C-terminal	4.31	1.37 × 10^−13^
AT2G31880	SOBIR1	SUPPRESSOR OF BIR1 1	−1.39	5.66 × 10^−4^
AT2G46240	BAG6	BCL-2-associated athanogene 6	4.71	2.42 × 10^−22^
AT3G01420	DOX1	Peroxidase superfamily protein	−0.30	8.85 × 10^−1^
AT3G06420	ATG8H	Autophagy 8h	1.22	7.54 × 10^−8^
AT3G06490	MYB108	Myb domain protein 108	2.43	1.86 × 10^−6^
AT3G09640	APX2	Ascorbate peroxidase 2	9.21	9.02 × 10^−18^
AT3G10525	LGO	LOSS OF GIANT CELLS FROM ORGANS	1.07	2.08 × 10^−7^
AT3G15352	COX17	Cytochrome c oxidase 17	1.63	3.35 × 10^−15^
AT3G16770	EBP	Ethylene-responsive element-binding protein	−0.83	2.07 × 10^−2^
AT3G44310	NIT1	Nitrilase 1	1.25	6.48 × 10^−42^
AT3G52400	SYP122	Syntaxin of plants 122	−1.30	1.63 × 10^−2^
AT4G00020	BRCA2(IV)	BREAST CANCER 2-like 2A	−1.42	5.46 × 10^−5^
AT4G14400	ACD6	ACCELERATED CELL DEATH 6	−1.01	2.58 × 10^−3^
AT4G19700	RING	SBP (S-ribonuclease -binding protein) family protein	1.14	2.52 × 10^−18^
AT4G23210	CRK13	Cysteine-rich RLK (RECEPTOR-like protein kinase) 13	1.37	2.63 × 10^−6^
AT4G25110	MC2	Metacaspase 2	−1.25	5.41 × 10^−5^
AT4G32940	GAMMA-VPE	Gamma vacuolar processing enzyme	−0.57	2.82 × 10^−1^
AT4G34410	RRTF1	Redox responsive transcription factor 1	−1.12	4.39 × 10^−1^
AT5G01630	BRCA2B	BRCA2-like B	−1.22	2.88 × 10^−3^
AT5G02190	PCS1	PROMOTION OF CELL SURVIVAL 1	−1.55	2.97 × 10^−3^
AT5G10380	RING1	RING/U-box superfamily protein	−1.28	6.18 × 10^−5^
AT5G26340	MSS1	Major facilitator superfamily protein	1.20	4.13 × 10^−3^
AT5G50260	CEP1	Cysteine endopeptidase 1	1.99	7.10 × 10^−3^

**Table 5:** The DEGs assigned to HSPs, HSFs, and their related genes. The aforementioned genes are primarily comprised of heat shock proteins, DNAJ heat shock proteins, heat shock transcription factors, and chaperones. The genes are classified according to their annotation, function, and the results of the following analyses: GO enrichment, MapMan, DAVID, and KEGG.

GeneID	Gene Symbol	Gene Name	Log2FC (Flash Treatment)	FDR (Flash Treatment)
AT1G53540	AT1G53540	HSP20-like chaperones superfamily protein	13.70	5.27 × 10^−43^
AT4G10250	ATHSP22.0	HSP20-like chaperones superfamily protein	13.23	8.52 × 10^−35^
AT4G25200	HSP23.6-MITO	mitochondrion-localized small heat shock protein 23.6	12.30	4.29 × 10^−29^
AT5G12020	HSP17.6II	17.6 kDa class II heat shock protein	12.01	1.95 × 10^−28^
AT3G46230	HSP17.4	Heat shock protein 17.4	11.46	4.78 × 10^−78^
AT4G27670	HSP21	Heat shock protein 21	11.13	6.69 × 10^−33^
AT5G12030	HSP17.6A	Heat shock protein 17.6A	11.02	1.67 × 10^−32^
AT2G29500	AT2G29500	HSP20-like chaperones superfamily protein	9.09	8.85 × 10^−80^
AT1G07400	AT1G07400	HSP20-like chaperones superfamily protein	8.22	1.12 × 10^−42^
AT5G59720	HSP18.2	Heat shock protein 18.2	7.47	5.91 × 10^−115^
AT1G74310	HSP101	Heat shock protein 101	7.44	1.64 × 10^−45^
AT5G51440	AT5G51440	HSP20-like chaperones superfamily protein	7.11	1.88 × 10^−38^
AT1G16030	Hsp70b	Heat shock protein 70B	7.04	8.58 × 10^−35^
AT1G54050	AT1G54050	HSP20-like chaperones superfamily protein	6.43	2.41 × 10^−25^
AT5G52640	HSP90.1	Heat shock protein 90.1	6.30	1.47 × 10^−30^
AT3G12580	HSP70	Heat shock protein 70	5.82	1.11 × 10^−29^
AT4G27890	AT4G27890	HSP20-like chaperones superfamily protein	5.79	1.50 × 10^−6^
AT1G52560	AT1G52560	HSP20-like chaperones superfamily protein	5.69	5.12 × 10^−23^
AT2G32120	HSP70T-2	Heat-shock protein 70T-2	5.46	8.53 × 10^−39^
AT2G26150	HSFA2	Heat shock transcription factor A2	5.24	9.11 × 10^−20^
AT1G59860	AT1G59860	HSP20-like chaperones superfamily protein	5.03	9.21 × 10^−16^
AT5G03720	HSFA3	Heat shock transcription factor A3	4.30	1.39 × 10^−12^
AT5G37670	AT5G37670	HSP20-like chaperones superfamily protein	3.90	5.52 × 10^−29^
AT2G20560	AT2G20560	DNAJ heat shock family protein	3.75	2.50 × 10^−15^
AT3G09350	Fes1A	Fes1A	3.73	1.31 × 10^−15^
AT2G03020	AT2G03020	Heat shock protein HSP20/alpha crystallin family	2.82	2.22 × 10^−3^
AT3G08970	ATERDJ3A	DNAJ heat shock N-terminal domain-containing protein	2.32	4.33 × 10^−12^
AT3G09440	AT3G09440	Heat shock protein 70 (Hsp 70) family protein	2.21	9.47 × 10^−7^
AT2G25140	CLPB4	Casein lytic proteinase B4	1.95	8.09 × 10^−12^
AT4G21870	AT4G21870	HSP20-like chaperones superfamily protein	1.89	7.23 × 10^−11^
AT5G23240	AT5G23240	DNAJ heat shock N-terminal domain-containing protein	1.87	1.86 × 10^−7^
AT4G37910	mtHsc70-1	Mitochondrial heat shock protein 70-1	1.81	1.45 × 10^−21^
AT4G32208	AT4G32208	Heat shock protein 70 (Hsp 70) family protein	1.73	7.91 × 10^−3^
AT3G51910	HSFA7A	heat shock transcription factor A7A	1.58	1.12 × 10^−2^
AT1G28210	ATJ1	DNAJ heat shock family protein	1.40	1.69 × 10^−15^
AT2G19310	AT2G19310	HSP20-like chaperones superfamily protein	1.38	1.36 × 10^−7^
AT3G25230	ROF1	Rotamase FKBP 1	1.20	8.91 × 10^−6^
AT5G02490	Hsp70-2	Heat shock protein 70 (Hsp 70) family protein	1.19	2.21 × 10^−3^
AT5G42020	BIP2	Heat shock protein 70 (Hsp 70) family protein	1.17	4.10 × 10^−6^
AT3G07770	Hsp89.1	Heat shock protein 89.1	1.16	4.65 × 10^−12^
AT5G56030	HSP81-2	Heat shock protein 81-2	1.16	9.10 × 10^−4^
AT5G28540	BIP1	Heat shock protein 70 (Hsp 70) family protein	1.15	2.05 × 10^−6^
AT2G33210	HSP60-2	Heat shock protein 60-2	1.12	6.76 × 10^−7^
AT1G79920	Hsp70-15	Heat shock protein 70-15	1.07	3.89 × 10^−6^
AT1G09080	BIP3	Binding protein 3	0.72	1.31 × 10^−1^
AT3G62600	ATERDJ3B	DNAJ heat shock family protein	0.60	1.33 × 10^−3^
AT5G43840	HSFA6A	Heat shock transcription factor A6A	0.02	9.87 × 10^−1^
AT5G47590	AT5G47590	Heat shock protein HSP20/alpha crystallin family	−0.79	2.92 × 10^−2^
AT2G20550	AT2G20550	HSP40/DnaJ peptide-binding protein	−1.11	2.75 × 10^−5^
AT5G18730	AT5G18730	DNAJ heat shock amino-terminal domain protein	−1.15	6.11 × 10^−3^
AT1G76780	AT1G76780	HSP20-like chaperones superfamily protein	−1.16	2.49 × 10^−3^
AT3G24520	HSFC1	Heat shock transcription factor C1	−1.24	5.53 × 10^−5^
AT5G20970	AT5G20970	HSP20-like chaperones superfamily protein	−1.26	7.48 × 10^−3^
AT3G10680	AT3G10680	HSP20-like chaperones superfamily protein	−1.27	3.88 × 10^−2^
AT1G75690	LQY1	Low quantum yield of photosystem II 1	−1.33	1.70 × 10^−6^

**Table 6:** The DEGs related to the cell cycle, division, and DNA replication and repair. In total, 71 DEGs were identified under these gene groups. The gene families were mainly MCM, Cyclin, UV-B-insensitive4, CDC, ORC, and SMR. The genes are classified according to their annotation, function, and the results of the following analyses: GO enrichment, MapMan, DAVID, and KEGG.

GeneID	Gene Symbol	Gene Name	Log2FC (Flash Treatment)	FDR (Flash Treatment)
AT1G03780	TPX2	Targeting protein for XKLP2	−2.19	7.11 × 10^−3^
AT1G07370	PCNA1	Proliferating cellular nuclear antigen 1	−1.33	8.32 × 10^−3^
AT1G08260	TIL1	TILTED 1	−1.19	6.70 × 10^−4^
AT1G08560	SYP111	Syntaxin of plants 111	−1.69	5.58 × 10^−4^
AT1G09450	Haspin	Haspin-related gene	−2.17	2.65 × 10^−3^
AT1G18800	NRP2	NAP1-related protein 2	1.03	7.74 × 10^−7^
AT1G20610	CYCB2;3	Cyclin B2;3	−1.13	1.43 × 10^−5^
AT1G44110	CYCA1;1	Cyclin A1;1	−2.25	8.35 × 10^−4^
AT1G44900	MCM2	Minichromosome maintenance 2	−1.64	1.57 × 10^−3^
AT1G53140	DRP5A	Dynamin related protein 5A	−2.11	4.65 × 10^−3^
AT1G67320	EMB2813	EMBRYO DEFECTIVE 2813	−1.09	3.46 × 10^−3^
AT1G70210	CYCD1;1	Cyclin D1;1	−1.46	1.73 × 10^−3^
AT1G73690	CDKD1;1	Cyclin-dependent kinase D1;1	1.46	1.85 × 10^−3^
AT1G76310	CYCB2;4	Cyclin B2;4	−1.94	2.78 × 10^−2^
AT1G76540	CDKB2;1	Cyclin-dependent kinase B2;1	−1.93	2.82 × 10^−4^
AT2G01120	ORC4	Origin recognition complex subunit 4	−1.05	1.48 × 10^−3^
AT2G07690	MCM5	Minichromosome maintenance 5	−1.26	1.43 × 10^−2^
AT2G16440	MCM4	Minichromosome maintenance 4	−1.37	1.75 × 10^−2^
AT2G20980	MCM10	Minichromosome maintenance 10	−2.36	1.39 × 10^−4^
AT2G25480	AT2G25480	TPX2 (targeting protein for Xklp2) protein family	−1.19	7.32 × 10^−6^
AT2G26760	CYCB1;4	Cyclin B1;4	−2.43	1.19 × 10^−2^
AT2G29680	CDC6	Cell division control 6	−1.94	6.83 × 10^−3^
AT2G37560	ORC2	Origin recognition complex second largest subunit 2	−1.65	1.01 × 10^−2^
AT2G39310	JAL22	Jacalin-related lectin 22	1.75	3.77 × 10^−2^
AT2G40550	ETG1	E2F target gene 1	−1.17	2.96 × 10^−2^
AT2G42220	AT2G42220	Rhodanese/Cell cycle control phosphatase superfamily protein	−1.56	4.73 × 10^−5^
AT2G42260	UVI4	UV-B-INSENSITIVE 4	−1.94	6.18 × 10^−3^
AT2G44580	AT2G44580	Zinc ion binding	−1.56	1.09 × 10^−2^
AT3G01710	AT3G01710	TPX2 (targeting protein for Xklp2) protein family	−1.40	4.08 × 10^−2^
AT3G02820	AT3G02820	Zinc knuckle (CCHC-type) family protein	−1.71	3.69 × 10^−5^
AT3G11520	CYCB1;3	Cyclin B1;3	−1.73	4.26 × 10^−2^
AT3G18730	TSK	TONSOKU	−1.38	1.08 × 10^−2^
AT3G25980	MAD2	MITOTIC ARREST-DEFICIENT 2	−1.56	2.61 × 10^−2^
AT3G26050	AT3G26050	TPX2 (targeting protein for Xklp2) protein family	−2.23	4.24 × 10^−2^
AT3G27630	AT3G27630	Cyclin-dependent kinase inhibitor SMR3-like protein	3.11	3.27 × 10^−2^
AT3G51280	AT3G51280	Tetratricopeptide repeat (TPR)-like superfamily protein	−2.73	1.01 × 10^−3^
AT3G53230	CDC48B	Cell division cycle 48B	2.18	5.55 × 10^−13^
AT3G54180	CDKB1;1	Cyclin-dependent kinase B1;1	−1.38	1.98 × 10^−2^
AT3G57860	UVI4-LIKE	UV-B-insensitive 4-like	−2.27	2.82 × 10^−3^
AT3G58650	TRM7	TON1 Recruiting Motif 7	−1.89	3.71 × 10^−2^
AT3G59550	SYN3	Rad21/Rec8-like family protein	−1.74	1.56 × 10^−2^
AT4G02060	PRL	PROLIFERA	−1.61	5.89 × 10^−3^
AT4G03210	XTH9	Xyloglucan endotransglucosylase/hydrolase 9	−1.07	7.33 × 10^−4^
AT4G03270	CYCD6;1	Cyclin D6;1	−1.52	1.70 × 10^−2^
AT4G05440	EDA35	Embryo sac development arrest 35	1.39	3.29 × 10^−10^
AT4G14310	AT4G14310	Transducin/WD40 repeat-like superfamily protein	−1.78	5.47 × 10^−4^
AT4G22860	AT4G22860	Cell cycle-regulated microtubule-associated protein	−2.52	2.66 × 10^−3^
AT4G22970	ESP	Homolog of separase	1.10	4.72 × 10^−4^
AT4G33260	CDC20.2	Cell division cycle 20.2	−1.65	9.16 × 10^−4^
AT4G33270	CDC20.1	Cell division cycle 20.1	−1.40	4.66 × 10^−2^
AT4G37490	CYCB1;1	Cyclin B1;1	0.28	8.05 × 10^−1^
AT5G02220	AT5G02220	SMR4 (SIAMESE/SIAMESE-RELATED)	−0.32	8.28 × 10^−1^
AT5G02420	AT5G02420	SMR3	−0.79	1.34 × 10^−1^
AT5G03270	LOG6	LONELY GUY 6	−2.07	8.95 × 10^−4^
AT5G06150	CYC1BAT	CYC1BAT, CYCB1;2	−1.76	3.83 × 10^−5^
AT5G06300	LOG7	LONELY GUY 7	−1.24	4.74 × 10^−3^
AT5G08020	RPA70B	RPA70-kDa subunit B	−1.37	1.97 × 10^−2^
AT5G10440	CYCD4;2	Cyclin d4;2	−1.03	2.29 × 10^−3^
AT5G13840	FZR3	FIZZY-related 3	−1.56	3.41 × 10^−4^
AT5G16690	ORC3	Origin recognition complex subunit 3	−1.46	5.77 × 10^−3^
AT5G40460	AT5G40460	Cyclin-dependent kinase inhibitor SMR3-like protein	1.86	1.01 × 10^−4^
AT5G43080	CYCA3;1	Cyclin A3;1	−1.15	4.93 × 10^−2^
AT5G44635	MCM6	Minichromosome maintenance 6	−1.37	1.15 × 10^−2^
AT5G46280	MCM3	Minichromosome maintenance 3	−1.39	2.78 × 10^−2^
AT5G48600	SMC3	Structural maintenance of chromosome 3	−1.25	4.81 × 10^−4^
AT5G48820	ICK6	Inhibitor/interactor with cyclin-dependent kinase	0.60	4.55 × 10^−1^
AT5G51600	PLE	PLEIADE	−1.76	6.98 × 10^−3^
AT5G61000	RPA70D	Replication factor-A protein 1-related	−1.89	1.33 × 10^−4^
AT5G67060	HEC1	HECATE 1	−2.67	1.32 × 10^−2^
AT5G67100	ICU2	INCURVATA2	−1.42	1.15 × 10^−3^
AT5G67270	EB1C	End binding protein 1C	−2.23	6.38 × 10^−4^

## Data Availability

The data are available upon request from the corresponding author.

## Supplementary Materials

The following supporting information can be downloaded at: https://www.mdpi.com/article/10.3390/ijms252413718/s1.
