# Supplementary material for: Comparison of the Effects of UV-C Light in the Form of Flash or Continuous Exposure: A Transcriptomic Analysis on Arabidopsis thaliana L
Source: Int J Mol Sci. 2024 Dec 22;25(24):13718. doi: 10.3390/ijms252413718 (PMC11676349; doi:10.3390/ijms252413718)
Supplement: Supplementary file 1 [file ijms-25-13718-s001.zip › Supplementary_Figure.pdf]

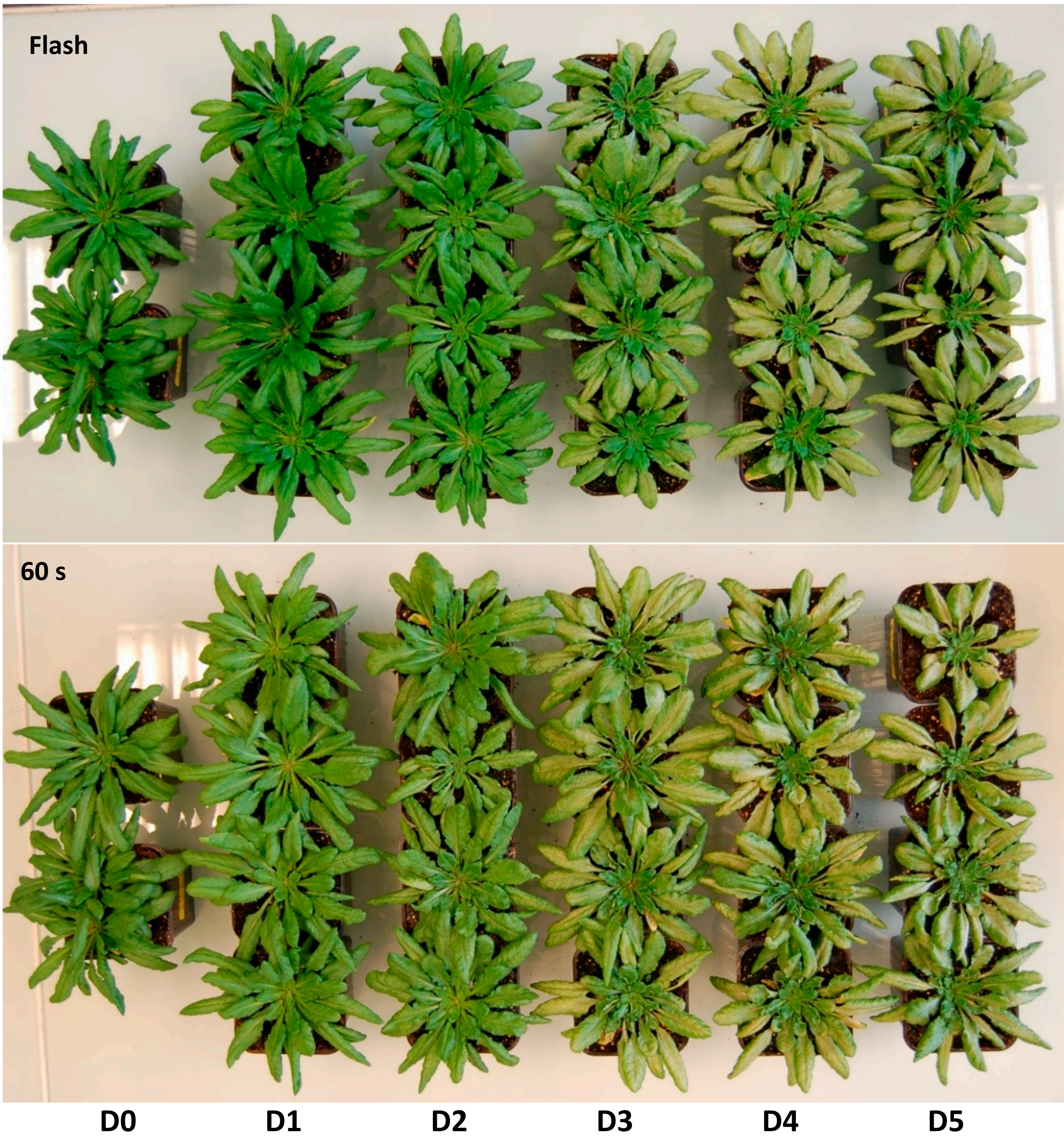

Figure S1. The selection of non-harmful doses of UV-C radiation on Arabidopsis plants. **Above**: Flash treatment. **Below**: 60 s treatment. The Arabidopsis plants were exposed to different dose of UV-C radiation in 254 nm in order to choose the appropriate dose with no phenotypically deleterious effects on plants. Five doses with 100 Jm^{-2} difference between two following doses were analyzed to determine which dose is suitable based on phenotypic symptoms on leaves and also the previous publications. The D2 dose with 200 Jm^{-2} was selected as no color changes and no necrosis was detected for the flash treated plants (D2 of the above photo). D0= control (no-UV-C radiation), D1= 100 Jm^{-2}, D2= 200 Jm^{-2}, D3= 300 Jm^{-2}, D4= 400 Jm^{-2}, D5= 500 Jm^{-2}.
